# Can the remuneration scheme of general practitioners affect their antibiotic prescription behaviour?

**DOI:** 10.1186/s13561-026-00736-w

**Published:** 2026-02-06

**Authors:** Yana V. Zykova, Eivor H. Hoff, Kristian B. Kraft, Arnstein Mykletun, Kristian A. Østby

**Affiliations:** 1 Cluster for Health Service Research, Norwegian Institute of Public Health, Oslo, Norway; 2 School of Business and Economics, UiT The Arctic University of Norway, Tromsø, Norway; 3 Department of Community Medicine, UiT The Arctic University of Norway, Tromsø, Norway; 4 Office of the Auditor General of Norway, Oslo, Norway; 5 Institute of Health and Society, University of Oslo, Oslo, Norway; 6 Centre for Work and Mental Health, Nordland Hospital Trust, Bodø, Norway; 7 Centre for Population Health, Haukeland University Hospital, Bergen, Norway

**Keywords:** Fixed salary, Fee-for-service, Capitation, Reimbursement, Competition, Primary care, Economic incentives, Antibiotic resistance, I11, I18, J33

## Abstract

**Background:**

Antibiotic resistance poses a significant global health threat, exacerbated by over-prescription of antibiotics, which often happens in primary care for respiratory tract infections (RTIs). Studies indicate that up to half of these prescriptions may be unnecessary. Little is known about how general practitioners’ (GPs) remuneration schemes influence prescribing. GPs compensated via fee-for-service (FFS) and capitation (CAP) may face stronger incentives to prescribe antibiotics compared to salaried GPs, as prescriptions can signal quality, reduce consultation time, and aid patient retention — critical where reimbursements depend on consultations and list size. This study examines how GP remuneration influences antibiotic prescribing for RTIs using Norwegian register data.

**Methods:**

We utilized linked registry data (2015–2019) from the Control and Payment of Health Reimbursements Database (KUHR), National GP Registry, Norwegian Prescribed Drug Registry (NorPD), and Statistics Norway. We matched antibiotic prescriptions to patient-GP contacts for RTIs. The dataset covers regular GPs (mixed FFS/CAP or fixed salary) and locum GPs (FFS or salary). Outcomes: (1) probability of antibiotic prescription during RTI contacts; (2) probability of selecting non-phenoxymethylpenicillin (non-PcV). We used linear probability, logit, and probit models, controlling for GP, patient, contact, and practice attributes. To mitigate selection bias, we exploited within-GP variation among those who switched remuneration type during the study period.

**Results:**

Regular FFS/CAP GPs had a 12–15% higher relative probability of prescribing antibiotics for RTIs than salaried GPs, especially at initial contacts. When prescribing, they were 9–11% more likely to choose non-PcV. Switchers analyses showed that FFS/CAP increased prescription rates by 14% and non-PcV choice by 8%. Among locums, we did not find any significant difference in overall prescription rates between FFS and salary, but FFS locums favoured non-PcV by 7%.

**Conclusions:**

Remuneration schemes may influence antibiotic prescribing behaviour. FFS/CAP is linked to higher prescription rates and broader-spectrum antibiotic use among regular GPs, likely due to patient retention and time-efficiency incentives. Policy interventions, such as monitoring of prescriptions depending on the remuneration type or adjustments to the remuneration scheme (e.g., antibiotic-related pay-for-performance), could promote prudent prescribing. Further research is needed on prescription appropriateness and quality impacts.

**Supplementary Information:**

The online version contains supplementary material available at 10.1186/s13561-026-00736-w.

## Background

Excessive use of antibiotics worldwide has made antibiotic resistance (AR) significant global public health problem. Antibiotics currently used for treating infectious diseases have become steadily less effective. The development of new drugs has also slowed down dramatically due to economic and regulatory disincentives [1]. Appropriate use of existing antibiotics is, therefore, crucial. However, antibiotics are sometimes prescribed when such treatment has no or only a marginal effect. Respiratory tract infections (RTIs) constitute a large share of all infections treated in primary care [2], many of which are either viral or self-limiting bacterial infections [3, 4]. For example, according to Fleming-Dutra et al. [5], almost half of antibiotics prescribed for RTIs in the US were unnecessary. Thus, to limit the overuse of antibiotics, it is important to study factors that may cause unnecessary prescriptions. This paper focuses on the effect of general practitioners’ (GPs’) remuneration scheme on their decisions about antibiotic treatment of RTIs. We use nationwide rich Norwegian register data for our analyses. Even though Norway has relatively low antibiotic use compared to other OECD countries [6], not every governmental goal of antibiotic reduction has yet been met [7].

The most common types of remuneration schemes used for primary care providers worldwide are salary, capitation (CAP), fee-for-service (FFS), and a mixture of them [8]. Salaried physicians receive a fixed amount for working a certain number of hours annually. Compared to GPs working under CAP, who receive payment according to the number of registered patients, they usually have no financial incentives to increase the number of patients or provide shorter consultations. Under FFS, GPs are paid for the number of consultations and specific procedures, which motivates GPs to provide more services to the patients. Therefore, FFS is usually mixed with CAP to avoid overprovision of the services. The remuneration scheme is a powerful tool used by policymakers to influence GPs’ behaviour to achieve certain policy goals aimed at improving the quality of care.

Previous research on the effects of remuneration schemes on GPs’ behaviour, medical decision-making is broad [8–12] and includes literature on the Norwegian market. For example, Brekke et al. [13] found that a higher consultation fee in an FFS system increased the number of visits but reduced the treatment intensity. In a later study by [14], it was found that when GP locums were reimbursed by FFS, they were more prone to charge more services for their patients (e.g., prolonged consultations, recall visits, laboratory tests) as well as more likely to issue sickness certificates, compared to when they were salaried. This effect was found to be stronger for more profit-oriented providers. Iversen and Lurås [15] also predicted that the CAP component in GPs’ remuneration scheme increased the rate of referrals to specialists. Further, Sørensen and Grytten [16] found that GPs with FFS contracts provided more consultations and other patient contacts than those paid a fixed salary. However, Grytten and Sørensen [17] found no difference in how these two groups of GPs respond to the increased competition with the service provision. In contrast to this study, Brekke et al. [18] found a positive effect of the competitive environment on the provision of sickness certificates by GPs with an activity-based contract.

Similarly, the remuneration scheme may affect antibiotic prescription decisions. Patients may request antibiotics for viral or self-limiting bacterial infection or consider antibiotic prescription (and/or eventual rapid resolution of the symptoms) as a quality-of-care mark. Patients may also dislike “wait-and-see” due to potential risks in case of diagnostic uncertainty and follow-up visits, which may imply out-of-pocket costs. While patient demand and copays apply across schemes, the risks of denying patients for GPs paid by FFS and/or CAP differ from salaried GPs. Under FFS/CAP, patient dissatisfaction may reduce income via smaller lists or fewer consultations in the future. Although follow-up appointments are billable under FFS and may generate additional profit, immediate prescription may still be preferrable over “wait-and-see” strategy due to the following reasons. First, follow-ups are uncertain (patients may recover without returning or seek help from other providers. Second, denying antibiotic treatment in favour of “wait-and-see” strategy may compromise the doctor-patient relationship, leading to patients seeking medical care from other providers. Finally, denying immediate treatment can be time-consuming [19] because it often requires extended explanations about the self-limiting nature of the infection, risks of unnecessary antibiotics (including AR), symptom management at home, and instructions on when to return if symptoms worsen. For GPs receiving FFS, immediate prescribing shortens visits, freeing capacity for additional revenue-generating consultations.

In Norway, regular self-employed GPs are remunerated via a mixed scheme (as described in the Institutional Setting section below), combining strong volume incentives (more consultations and procedures) with retention incentives (maintaining/enlarging the list). This dual structure may particularly encourage time-efficient solutions [16, 20] to balance higher number of consultations with list stability. In contrast, locums (who cover absent regular self-employed GPs’ lists) are typically paid only FFS without receiving a CAP component, meaning volume incentives dominate while direct retention benefits often accrue to the regular GP whose list they cover. This may somewhat weaken incentives to prescribe antibiotics as a retention tool compared to regular mixed-scheme GPs. Unlike FFS/CAP, salaried GPs, typically municipally employed with fixed working hours, face no direct financial incentives tied to consultation volume or list size. They have greater discretion to allocate time within their scheduled office hours to longer, guideline-adherent consultations, including watchful waiting strategies. Extending consultation time or accepting longer waiting lists for non-urgent cases can be self-regulating, as patients may switch GPs, and minor self-limiting conditions resolve spontaneously.

To our knowledge, there are only a few papers about the effect of remuneration schemes on antibiotic prescriptions. These papers primarily focus on antibiotic-related pay-for-performance indicators and find that antibiotic prescription behaviour improves when a provider is paid for achieving specific antibiotic-related performance measures in addition to their main remuneration scheme [21, 22]. There is one paper about the effect of the main remuneration types on prescriptions. Hutchinson and Foley [23] found that in 1996 in Newfoundland, Canada, GPs paid by FFS had higher antibiotic prescription rates than their salaried counterparts. The rate was calculated as the mean number of antibiotic prescriptions per unique patient receiving such drugs per year for each GP. Their study provides important insight into the GPs’ prescription behaviour but suffers from several data limitations that do not allow addressing potential biases and, therefore, a better understanding of the underlying mechanisms of GPs’ behaviour. The study’s major limitation is the lack of data on the number of patient visits to GPs. However, providers paid by FFS may systematically prescribe more drugs to the patients because they might have a higher share of patients with diseases that require the prescription of antibiotics or cause more frequent infection episodes.

Our paper differs from the paper by Hutchinson and Foley [23] in several ways. First, we use nationwide register-based data, which allows us to test the following differences. The analysis of regular GPs allows us to study the difference between a mix of FFS and CAP and fixed salary, while the analysis of locum GPs allows us to study the difference between FFS and salary. Second, the individual characteristics of GPs working under different remuneration schemes may systematically differ. For example, if more profit-oriented GPs are more likely to choose to work under a certain remuneration scheme, the difference in prescriptions between different schemes may rather be explained by profit-maximizing preferences. Like Brekke et al. [14], we address this bias by studying the behaviour of GPs who switched between different remuneration schemes at least once during the study period.

Moreover, our data enables us to determine whether a prescribed antibiotic was broad- or narrow-spectrum. Therefore, we can test whether the remuneration scheme affects the choice of the drug when antibiotics are prescribed. The reason for such analysis is that broad-spectrum antibiotics are more effective against a wider range of bacteria species and, hence, to a larger extent, contribute to the spread of AR. Therefore, it is advisable to select narrow-spectrum antibiotics when possible. However, prescribing a broad-spectrum antibiotic can increase the likelihood of successful treatment, which patients may interpret as a sign of high-quality care. This approach may enable GPs to minimize patient dissatisfaction and reduce the time spent on a more detailed assessment of the type of infection and the guidelines and risks associated with selecting the incorrect medication.

Finally, we use individual-level data on antibiotic prescriptions combined with data about patient visits for RTIs, which allows us to address the following limitations. Previous research on the GPs’ prescription behaviour used the number of prescriptions or the quantity of drug (adjusted or non-adjusted for the number of listed patients) as a measure of doctors’ likelihood of providing medication [24–27]. This approach may suffer from several limitations. First, since patients may be treated by a different GP than the one, they are registered with, the analysis is often limited to the prescriptions to registered patients. Second, GPs working under different remuneration schemes may systematically vary in the clinical tasks they are exposed to due to differences in patient characteristics and needs. Moreover, some antibiotics used to treat RTIs can also be prescribed for other infectious diseases, e.g., urinary tract infections, skin infections, and sexually transmitted infections. Finally, and most importantly, GPs may work different numbers of hours and days in their practice. According to our data, about 50% of GPs at some point had a substitute doctor working for them at a certain percentage in the practice. One GP could have several substitute doctors simultaneously in the same month and can be a substitute doctor for another GP. At the same time, the data does not contain direct information about where the patient’s contact/prescription took place. Therefore, the number of antibiotic prescriptions per listed patient does not necessarily reflect a specific GP’s antibiotic prescription behaviour. We solve this problem by analysing patients’ contact with a GP for RTI. Regardless of the remuneration type, all GPs are obliged to register all patient contacts and the corresponding diagnosis [28]. We can also identify if the contact happened at the GP’s regular practice by excluding the days when that GP was registered as a treating doctor at several places. Additionally, we can determine whether multiple visits to a GP occurred during a single RTI episode. We can also find out if the GP prescribed an antibiotic during the first visit or a later visit, when the prescription was more likely to be necessary.

## Institutional background

Norwegian residents are covered by public health insurance, known as Folketrygden, which is funded through taxes and contributions from both employees and employers [29]. Patients’ out-of-pocket payments account for about 15% of the total health expenditure [30]. As of 2025, the basic fee for a consultation with a GP was 179 NOK (about 15 euros), which increased to 235 NOK (about 20 euros) if the GP holds a specialization in general medicine. Certain visits are exempt from this fee, for example, children under 16 years of age, visits related to prenatal care, and visits related to transmittable diseases that pose a threat to public health [31]. Once a patient reaches a certain limit on fees, they are exempt from further co-payments for the rest of the year [32]. The maximum user fee in 2025 was 3,278 NOK (about 280 euros) [31].

The primary healthcare system in Norway is managed and organized by municipalities, which enter contracts with GPs. GPs play a crucial role in the Norwegian healthcare system due to their strong gatekeeping function, where they handle all initial assessments, treatments, and referrals to secondary care [32]. In October 2023, there were 5224 GPs in Norway [33].

In 2001, Norway introduced a reform called The Regular General Practitioners Scheme (the ‘list-patient’ system), allowing patients to choose their regular GP (given available spots on the list) and change their GP twice a year. GPs were not allowed to choose which patients entered their lists but could determine the maximum length of their patient list within an interval between 500 and 2500 patients [18]. In 2017, the average list length was 1157 patients [34].

Although the central government still primarily funds and regulates primary healthcare, the market has become more competitive [18] mainly due to the free choice of GP and the fact that most GPs in Norway are self-employed (about 85%, according to our data) and reimbursed by a mix of FFS , CAP, and co-payments from patients. During the study period, CAP constituted around 30% of the GP earnings. The remaining 15% of GPs are salaried and are employed by the municipalities [32]. Salaried GPs also register the fees for the services provided; however, this fee income is paid to the municipalities. In 2019, around half of the salaried GPs had special agreements with the municipalities [35], where GPs typically receive 20–30% of the FFS earnings [36].^1^ The level of salary does not directly depend on the actual list size but might reflect the lower limit of the preferred list length if stated in the contract [14].

Self-employed GPs are responsible for managing and organizing their practice (usually with other GPs) and providing necessary services to their listed patients within a reasonable time. A typical practice comprises a group of two to six GPs and supporting staff [32]. A full-time regular GP responsible for the list is obliged to be available at least 28 h a week and 44 weeks a year with some exemptions (e.g., sickness or parental leave). In case of shorter absences (e.g., due to vacation, courses, or holidays), their colleagues can cover for them. However, in many cases (especially relevant for longer absences), a temporary substitute doctor (locum) is needed. Locums’ remuneration scheme is mirrored by the corresponding regular GP’s remuneration, except that locums do not receive a CAP component [14].

Municipalities are also responsible for organizing emergency care, represented by local primary care emergency centres (PCECs), which provide care on weekends, evenings, and nights^2^. Regular GPs are usually obliged to work at such centres unless they are exempted from this, e.g. due to age, health, or social reasons, or if the municipality has hired permanent doctors to work at PCECs [14]. Moreover, a certain number of hours (minimum 240) of work at PCECs is required for obtaining a specialization in general medicine [37]. Specialization in general medicine allows GPs to increase their earnings due to higher patient co-payment and a special fee for being a specialist, which gave a GP an extra 120 NOK (about 10 euros) for a standard consultation in 2025 [38].

## Data

### Data sources and variables

To test the differences in GPs’ behaviour under different remuneration schemes, we used data from several nationwide registries linked together. In our study, we included all Norwegian GPs (regular and locums) and their treated patients for 2015–2019. Data sources include the Control and Payment of Health Reimbursements Database (KUHR), the National GP Registry (Fastlegeregisteret), the Norwegian Prescribed Drug Registry (NorPD), and Statistics Norway (SSB). The KUHR database contains all reimbursement claims from practicing GPs. For each reimbursement claim, we have a GP and a patient identifier, the GP’s remuneration type, as well as information about the date, time, type of the GP-patient contact, registered fees, amount claimed, patient co-payment, and the contact’s associated RTI diagnosis, if any, according to the International Classification of Primary Care (ICPC-2). From the GP Registry, we got monthly event-history data on GP contracts, including the GP’s contracted role, desired list length, current list occupancy, anonymized practice municipality ID, and monthly event-history data on patients listed with different regular GP contracts during the period. From SSB, we obtained information on patients’ birth year, gender, household income, and pseudonymized municipality. From NorPD, we got electronic records of all prescription drugs dispensed by Norwegian pharmacies.

### Antibiotic prescriptions and RTI contacts

From NorPD data, we selected all prescriptions of antibiotics that belonged to the group ‘J01 – Antibacterials for systemic use’ according to Anatomical Therapeutic Chemical classification system (ATC). We excluded methenamine (J01XX05), nitrofurantoin (J01XE01), (piv)mecillinam (J01CA08) and trimethoprim (J01EA01). These drugs have been excluded because, in Norway, they are exclusively used for urinary tract infections [39].

From KUHR, we selected all patients’ contacts with GPs for RTI-related diagnoses based on the relevant ICPC-2 code and combined the diagnoses in groups, as presented in Table 1,^3^. Similarly to the previous studies on RTI episodes [39–42], we distinguished between single RTI contacts and RTI episodes. This distinction is important since patients may have several visits to GPs during one RTI episode and get more antibiotic prescriptions due to the previous antibiotics prescribed being inefficient against specific bacteria species. It may also be important to examine GPs’ prescription behaviour during the first/only contact of an RTI episode to understand if the GPs apply a “wait-and-see” strategy or prescribe an antibiotic treatment immediately. Further, we present the analysis on the primary dataset and the datasets, including the first (the only) contacts during infection episodes^4^. Around 67% of all contacts for RTIs from the dataset were the first or the only contact during an RTI episode. The information about each GP contact/consultation from KUHR cannot be directly linked to a prescription from NorPD. Therefore, we matched this data using an approach similar to one previously used in the literature about antibiotic treatment of RTIs in Norway [39, 43]. We matched the contacts to the prescription data from NorPD by a combination of date, patient ID, and GP ID. A contact made 0–7 days before the prescription was considered as the contact leading to the prescription^5^. About 95% of matched antibiotic prescriptions have been collected by the patient within the first three days after contact with the GP.

**Table 1:** Diagnoses codes included in the analysis

Diagnoses	ICPC-2 codes
Upper respiratory tract symptoms and infections	R01-R29, R74
Acute bronchitis	R78
Chronic bronchitis/ COPD	R79, R95
Ear infection	H71, H72, H74
Pneumonia	R81
Sinusitis	R75
Acute tonsilitis	R72/76
Other respiratory diagnoses	Other R diagnoses except R96 and R97

ICPC-2 codes – diagnoses codes according to the International Classification of Primary Care

### Outcome variables

To study GPs’ antibiotic prescription behaviour, we create two outcome variables. 

#### Outcome variable 1

To measure the relationship between the remuneration scheme and the probability of prescribing an antibiotic for RTI, we use a binary outcome variable which is equal to one if the contact has been matched to an antibiotic prescription from the NorPD dataset, and zero otherwise^6^.

#### Outcome variable 2

To examine whether there is a relationship between the choice of antibiotic for RTI and the GP remuneration scheme, we use the following outcome variable. According to the current national guidelines, a narrow-spectrum antibiotic phenoxymethylpenicillin (PcV) (J01CE02) is recommended as the first-choice drug for antibiotic treatment of almost all RTIs [44]. Therefore, for all contacts leading to an antibiotic prescription, we create a binary variable equal to 1 if a non-PcV antibiotic has been chosen and zero otherwise^7^.

### Main datasets and subpopulation definitions

We define two main GP populations for which we construct separate datasets: regular GPs and GP locums. Regular GPs are defined as all GPs that have been registered as the owner of a contract during the study period. During some short time spans within the study period, about 5.6% of all regular GPs were at the same time registered as locums on other contracts or as regular GPs on two different contracts. Since we do not know where the contacts happened, we excluded those days from the analysis (less than 1% of observations excluded). The remaining activity data is then linked with data on list characteristics. We removed about 6% of observations due to missing patient, GP, or list characteristics data. The final dataset contains about 10 million observations.

GP locums are defined as any GP who has not been registered as the contacting patient’s regular GP or as a regular GP on any other contract that day. Again, we cannot perfectly identify the GP practice where the contact happened since locums may work on several contracts at the same time. Therefore, similarly to Brekke et al. [14], we link the data on patients’ contacts with the practice characteristics of the patient’s regular GP. Less than 4% of the data was missing and therefore excluded. The resulting dataset consists of about 1.5 million observations.

## Empirical approach

To study the difference in antibiotic prescription behaviour between different remuneration types, we use the following Eq. (1)

$$\begin{aligned} {y}_{\mathrm{itm}}=&\, {\upbeta}_{0}+{\upbeta}_{1}\mathrm{FFS}\_\mathrm{CAP}_{\mathrm{itm}}+{\upbeta}_{2}\mathrm{X}_{\mathrm{itm}}+{\upbeta}_{3}\mathrm{P}_{\mathrm{itm}}\\ &+{\upbeta}_{4}\mathrm{C}_{\mathrm{itm}}+{\upbeta}_{5}\mathrm{Y}_{\mathrm{itm}}+{\delta}_{\mathrm{m}}+{\eta}_{\mathrm{t}}+{\epsilon}_{\mathrm{itm}},\end{aligned}$$

where $\:{y}_{itm}$ measures the decision about antibiotic prescription of GP *i* in an RTI contact at time *t* in municipality *m; FFS_CAP* is a dummy variable equal to zero if a GP is paid a fixed salary, and one if a GP is paid by FFS or a mix of FFS and CAP; $\:{X}_{itm}$ is a vector of GP characteristics (such as gender, age and specialization); $\:{P}_{itm}$ is a vector of patient characteristics (age, gender, household income^8^, and the dummy variable for whether the patient contacted their regular GP); $\:{C}_{itm}$ is a vector of contact characteristics (contact type and the reason for contact, i.e. the associated RTI diagnosis); $\:{Y}_{itm}$ is a vector of practice or aggregated patient list characteristics (list length, whether the list has available spots, whether the practice is a group practice, the proportion of females on the list, average age, and proportion of patients with low income^9^). In addition to this, we include fixed effects for 424 municipalities^10^$\:{\delta\:}_{m}$ and time-fixed effects $\:{\eta\:}_{t}$ in the model. Finally, $\:{\epsilon}_{itm}\:$is an error term.

The inclusion of the above-mentioned factors allows for controlling for possible systematic differences between GPs working under different remuneration schemes. For example, we include municipality-fixed effects to avoid potential biases related to differences in access to GPs or secondary care in a geographic location. GPs from different municipalities can also be exposed to different levels of local competition and other factors. The length of the list may be different across remuneration types, while longer lists may imply longer waiting time and fewer patients with less severe infections which do not require antibiotic treatment or require a narrow-spectrum antibiotic. The information about the excess capacity on the list reflects GPs’ willingness to attract more patients and, hence, GP’s availability and access to the GP. In addition, the demographic characteristics of the list may characterize how demanding the GP list is in general. Further, the decisions about antibiotic prescription may be affected by how well the GP knows the patient. Since GPs may not equally often work with the patients not listed with them, we controlled for this by including the corresponding dummy variable. Finally, we included patient characteristics, such as age and gender, that may be correlated with the patient’s health status, antibiotic prescribing practices, and demand for antibiotics [45].

### Model 1 for the effect of remuneration scheme on the probability of antibiotic prescription

To measure the relationship between the remuneration scheme and the probability of prescribing an antibiotic for RTI, we estimate Eq. (1) separately for regular GPs and GP locums, with outcome variable 1. We make estimations based on the datasets mentioned in the data section, which include all RTI contacts, and on the datasets, which include only the first (or the only) contacts during each RTI episode.

Table 2 presents descriptive statistics for the datasets, which include all RTI contacts by the types of remuneration. The corresponding descriptive statistics for the datasets, including the first (or the only) contacts during RTI episodes, are presented in in Additional file 1 (Table S2). Table 2 shows that regular GPs paid by a mix of CAP and FFS prescribed antibiotics more often than those paid by salary, while the difference for locums is smaller and goes the opposite direction. GPs paid by FFS/CAP were specialists more often, regardless of whether the GP was regular or locum. The mean age for salaried GPs was 13 and 3 years lower that for GPs paid by FFS/CAP for regular GPs and locums, respectively. Locums were, on average, younger than regular GPs. For regular GPs, there are more observations for male GPs in the subset of those paid by FFS and CAP than in a subset of salaried GPS, while it is vice versa for locum GPs. Locums generally provide more consultations at the GP office than regular GPs. This share is slightly lower for salaried regular GPs compared to the regular GPs paid by FFS and CAP and is slightly higher for salaried locums compared to locums paid by FFS. Regular GPs paid by CAP/FFS provide consultations to the listed patients more often than salaried GPs. The lists are shorter and less often open for salaried GPs in both datasets. The average age of the listed patient, gender composition and proportion of patients with low income do not vary much with the remuneration type. Further, in the analysis, we will control for a wide range of variables related to the patient, GP, list and contact characteristics.

**Table 2:** Descriptive statistics for the datasets containing all RTI contacts decomposed by the remuneration types (Model 1)

	Regular GPs		Locums	
	FFS+CAP	Salary	FFS	Salary
Dependent variable				
Antibiotic prescriptions for RTI	14.69	11.95	15.01	15.43
GP characteristics				
Specialist	66.86	35.87	8.75	6.23
Male	65.23	51.94	48.71	59.94
Age	50.16 (10.89)	44.62 (11.39)	37.45 (9.95)	40.12 (13.76)
Patient characteristics				
Age group 0-2	6.28	6.11	7.83	6.96
Age group 3-6	5.28	4.79	6.19	5.48
Age group 7-12	3.97	3.48	4.33	4.02
Age group 13-18	6.80	6.00	7.44	7.32
Age group 19-25	7.44	7.94	8.61	8.73
Age group 26-45	21.64	18.12	24.16	18.97
Age group 46-65	25.46	24.10	22.75	22.85
Age group 66-85	20.67	25.56	16.64	22.34
Age group 86+	2.46	3.90	2.05	3.33
Male	43.51	44.02	42.34	44.89
Household income/100,000	3.67 (3.31)	3.39 (1.93)	3.66 (3.87)	3.42 (7.15)
Patient is listed with the GP	81.73	67.37		
Contact characteristics				
Contact type				
Simple encounter	16.55	17.14	11.39	10.88
Telephone contact	15.90	15.80	12.34	10.81
Consultation	67.23	66.60	76.03	77.90
Home visit	0.33	0.46	0.24	0.40
Diagnosis				
Upper RT symptoms and infections	49.76	49.99	55.98	54.34
Acute bronchitis	7.68	6.50	6.66	6.52
Chronic bronchitis/ COPD	10.46	13.12	6.89	9.09
Ear infection	3.73	3.67	4.02	4.05
Pneumonia	5.04	6.25	5.15	6.96
Sinusitis	5.41	4.71	5.23	5.03
Acute tonsilitis	3.12	2.96	3.58	3.86
Other respiratory diagnoses	14.80	12.78	12.50	10.14
Practice/list characteristics				
List length/100	13.04 (3.45)	8.66 (2.82)	12.52 (3.35)	9.16 (2.91)
Open list	20.28	15.63	16.74	14.27
Group practice	93.44	93.69	94.85	92.83
Proportion females	0.49 (0.10)	0.49 (0.09)	0.51 (0.10)	0.49 (0.08)
Average age	40.16 (5.38)	40.33 (6.47)	39.09 (5.63)	41.22 (6.13)
Proportion low income	0.18 (0.07)	0.20 (0.08)	0.18 (0.07)	0.20 (0.06)
Observations	9,532,662	445,606	1,370,521	149,674
Patients	2,554,971	177,554	703,961	91,062
GPs	5,217	1,020	3,575	1,412

Percentages are reported for the discrete variables; mean (standard deviation) – for continuous variables

### Model 2 for the effect of remuneration scheme on the choice of antibiotic

To measure the relationship between the remuneration scheme and the probability of choosing a non-PcV antibiotic for RTI, we estimate Eq. (1) separately for regular GPs and GP locums, with outcome variable 2. We make estimations based on the datasets, including all RTI contacts leading to an antibiotic prescription and its subset, including only the first (or the only) contacts during each RTI episode. Descriptive statistics for the corresponding datasets are presented in Table 3 and are similar to Table 2 except that both salaried regular GPs and salaried locums prescribed fewer non-PcV antibiotics than those paid by FFS/CAP. The corresponding descriptive statistics for the datasets, including the first (or the only) contacts during RTI episodes, are presented in in Additional file 1 (Table S3).

**Table 3:** Descriptive statistics for the data including all contacts leading to antibiotic prescriptions and decomposed by the remuneration types (Model 2)

	Regular GPs		Locums	
	FFS+CAP	Salary	FFS	Salary
Dependent variable				
Non-PcV antibiotic	55.92	48.42	45.73	42.71
GP characteristics				
Specialist	66.33	35.92	9.42	6.41
Male	65.73	55.10	49.66	62.14
Age	50.62 (10.76)	44.83 (11.22)	38.45 (10.83)	41.79 (14.30)
Patient characteristics				
Age group 0-2	6.18	6.32	7.65	6.14
Age group 3-6	6.74	6.37	8.13	7.10
Age group 7-12	4.74	4.56	5.23	4.62
Age group 13-18	6.47	6.08	6.92	7.54
Age group 19-25	8.33	9.68	9.20	9.90
Age group 26-45	24.14	21.65	25.33	21.26
Age group 46-65	24.57	22.72	21.45	22.46
Age group 66-85	17.01	19.74	14.51	18.54
Age group 86+	1.82	2.88	1.56	2.44
Male	42.52	42.35	41.46	43.62
Household income/100,000	3.71 (3.50)	3.47 (2.14)	3.69 (2.85)	3.51 (2.00)
Patient is listed with the GP	75.39	56.29		
Contact characteristics				
Contact type				
Simple encounter	5.15	5.20	3.08	2.95
Telephone contact	5.59	6.90	5.12	4.48
Consultation	88.95	87.4	91.57	92.25
Home visit	0.31	0.41	0.23	0.32
Diagnosis				
Upper RT symptoms and infections	28.03	24.91	30.07	26.56
Acute bronchitis	13.37	10.51	9.31	9.67
Chronic bronchitis/ COPD	4.17	5.90	2.96	3.89
Ear infection	8.17	10.26	10.82	10.50
Pneumonia	11.37	15.77	13.44	17.93
Sinusitis	14.21	13.65	12.21	12.51
Acute tonsilitis	11.77	13.05	14.59	14.83
Other respiratory diagnoses	8.17	5.94	6.59	4.11
Practice/list characteristics				
List length/100	13.21 (3.49)	8.85 (2.95)	12.57 (3.35)	9.43 (2.95)
Open list	21.08	17.09	17.48	15.26
Group practice	92.57	94.55	95.06	94.21
Proportion females	0.49 (0.10)	0.49 (0.09)	0.50 (0.10)	0.49 (0.08)
Average age	40.13 (5.33)	39.62 (6.41)	39.12 (5.60)	41.04 (5.99)
Proportion low income	0.18 (0.07)	0.20 (0.08)	0.18 (0.07)	0.19 (0.06)
Observations	1,432,738	54,571	210,959	23,655
Patients	904,738	41,293	174,813	20,515
GPs	5,192	967	3,258	1,205

Percentages are reported for the discrete variables; mean (standard deviation) – for continuous variables

### Models for the GPs switching the remuneration scheme

In this section, we provide additional analyses that attempt to address the following selection bias: If we find a positive effect of the FFS/CAP on prescriptions, it may be explained by the differences in the remuneration scheme. However, it may also be explained by the differences in GPs preferences if, e.g., more profit-oriented GPs systematically choose to work under a certain remuneration scheme. To address this bias, we use an approach similar to one previously used by Brekke et al. [14]. Separately for regular GPs and locum GPs, we select those GPs who have worked under both remuneration schemes during the study period and estimate Eq. (1) with GP fixed effects. Including GP fixed effects in the model allows for control for GP-related unobserved factors (including profit-maximizing preferences), i.e., to test the pure effect of the remuneration scheme within subjects. Such analysis assumes GPs’ preferences and practice styles are stable over time. This assumption is in line with a Norwegian study by Grytten and Sørensen [46], which concluded that GP practice style does not change with changes in patient population by studying the GPs who moved from one municipality to another. As regards the study of locums, Brekke et al. [14] argue that the subset of locums is highly representative of the general population of the Norwegian GPs since a high share of GPs (80% according to their data) starting their regular practice have worked as locums before. At the same time, it is also common for locums to work under different remuneration schemes since such choices are more affected by the availability of positions rather than profit-maximizing preferences [14]. The same logic does not apply to the regular GPs switching between different payment systems; however, in our data, we did not find any systematic pattern when the GPs first worked under one type of contract and then switched to another. Further, we provide separate analyses for the regular GPs and locums.

#### Regular GPs

The dataset of regular GPs contains some observations (less than 1% of total observations) when the data on the remuneration scheme according to the GP register did not correspond to the data on the remuneration scheme in KUHR. We do not know the exact reason for this, but from the data, it looks like those GPs worked on some other contract in addition to their primary contract, even though it is not reflected in the GP registry. Unfortunately, we are unaware of how many similar observations we have in the data when the remuneration scheme was the same in both registries. Most likely, such observations are rare since, according to the GP register, only about 5.6% of regular GPs additionally worked as locums on other contracts for different time spans during the study period. To check whether this issue affects the estimation results of the models in the previous section, we analysed the subset of contacts made by patients with their regular GP. Such selection allowed us to include the contacts which certainly happened at the GP regular practice. The analysis (available upon request) showed that the results are not affected by whether we include all contacts or only those with the listed patients.

Even though the above-mentioned issue does not affect the results of the models in previous sections, it may have an impact on the analysis of regular GPs who switched remuneration schemes because the number of GPs who have ever worked as regular GPs under different remuneration schemes is low compared to the general sample. Therefore, we base the later analysis purely on the regular GP contacts with their registered patients. According to our data, there are 177 GPs who have ever worked both as a salaried regular GP and a regular GP paid by a mix of FFS and CAP. About 59% of them are male (57% for non-switchers), while an average list length at the first observation in time was 975 patients. About 46% of switchers were salaried regular GPs at the first observation, and 55% of switchers were salaried at the last observation in time. The mean age for switchers and non-switchers is about 47.

#### Locums

According to our data, 563 GPs switched remuneration schemes at least once while being locums. About 60% of switchers are males, which is 10% more than among non-switchers. On average, switchers are about 40 years old, and they are about 2 years older than non-switchers. About 48% of switchers worked under FFS contracts at the beginning of the observation period and about 77% worked under FFS at their last observation. The mean list length at the first observation for each GP was 1023 patients, and 1118 patients at the last observation.

#### Descriptive statistics and modelling

In Table 4, we present the descriptive statistics for switchers (both regular GPs and locums) decomposed by the remuneration scheme for the datasets, including contacts leading and not leading to the prescription of antibiotics. Table 4 shows a similar pattern as the descriptive statistics for the general GP population (Table 2) with some exceptions. The difference in antibiotic prescription rates between remuneration schemes is lower for regular switchers than for the general population of regular GPs. Moreover, switchers, regardless of being regular GP or locum, working under different payment schemes are more similar to each other in terms of specialization and the length of the patient list than non-switchers. The household income of the patients of switchers is slightly lower than for non-switchers. Finally, the lists of regular GPs working under FFS and CAP who switched remuneration type are less often open than the lists of all other GPs.

**Table 4:** Descriptive statistics for GPs who switched remuneration type, datasets including all RTI contacts

	Regular GPs		Locums	
	FFS+CAP	Salary	FFS	Salary
Dependent variable				
Antibiotic prescriptions for RTI	12.50	11.80	15.96	17.02
GP characteristics				
Specialist	56.01	48.95	6.40	5.73
Male	58.27	62.66	57.28	62.60
Age	45.81 (10.36)	48.17 (11.82)	38.75 (10.85)	41.45 (13.18)
Patient characteristics				
Age group 0-2	5.53	4.90	7.74	6.95
Age group 3-6	4.68	4.70	6.20	5.75
Age group 7-12	3.61	3.39	4.47	4.15
Age group 13-18	5.46	5.83	7.47	7.48
Age group 19-25	6.27	7.02	8.38	8.88
Age group 26-45	19.18	17.46	23.73	19.12
Age group 46-65	26.51	26.24	22.77	22.74
Age group 66-85	25.58	26.66	17.19	21.73
Age group 86+	3.19	3.80	2.03	3.21
Male	44.54	44.21	43.61	44.68
Household income/100,000	3.45 (2.34)	3.37 (1.73)	3.56 (6.59)	3.40 (1.92)
Contact characteristics				
Contact type				
Simple encounter	18.08	19.52	11.09	10.35
Telephone contact	19.88	17.97	10.87	9.76
Consultation	61.43	62.03	77.80	79.52
Home visit	0.60	0.49	0.24	0.37
Diagnosis				
Upper RT symptoms and infections	49.31	48.72	56.50	55.27
Acute bronchitis	6.82	6.31	6.75	6.73
Chronic bronchitis/ COPD	14.21	14.48	7.19	8.77
Ear infection	3.34	3.51	3.90	3.85
Pneumonia	5.40	6.08	5.59	6.73
Sinusitis	5.17	4.88	5.23	5.20
Acute tonsilitis	2.59	2.31	3.72	3.97
Other respiratory diagnoses	13.15	13.72	11.12	9.48
Practice/list characteristics				
List length/100	11.49 (2.87)	9.62 (2.89)	11.92 (3.39)	9.44 (3.07)
Open list	11.87	17.04	15.77	13.70
Group practice	86.67	93.69	94.62	92.57
Proportion females	0.49	0.49	0.50 (0.10)	0.49 (0.08)
Average age	40.60 (5.48)	40.58 (5.87)	39.18 (5.44)	41.28 (6.21)
Proportion low income	0.19 (0.06)	0.19 (0.06)	0.19 (0.08)	0.20 (0.06)
Observations	111,679	44,647	189,738	62,045
Patients	39,538	18,765	120,530	42,370
GPs	177	177	563	563

Percentages are reported for the discrete variables; mean (standard deviation) – for continuous variables

Table 5 shows descriptive statistics for switchers for the datasets, including only contacts where an antibiotic has been prescribed. These datasets are used to estimate the effect of the remuneration scheme on the probability of choosing non-PcV antibiotics. Table 5 shows similar patterns as Table 3 (descriptive statistics on the general population), except for salaried locums switching remuneration type prescribed more non-PcV antibiotics, in contrast to less non-PcV antibiotics among salaried GPs in the general population of GPs.

**Table 5:** Descriptive statistics for GP who switched remuneration type, datasets including only contacts leading to an antibiotic prescription

	Regular GPs		Locums	
	FFS+CAP	Salary	FFS	Salary
Dependent variable				
Non-PcV antibiotic	56.18	55.01	43.92	46.30
GP characteristics				
Specialist	58.37	45.05	8.92	5.90
Male	57.21	61.39	59.47	66.19
Age	47.23 (10.66)	48.71 (11.54)	40.21 (11.75)	42.98 (13.92)
Patient characteristics				
Age group 0-2	5.54	5.07	7.02	5.31
Age group 3-6	5.94	5.90	7.80	7.05
Age group 7-12	4.69	4.00	5.02	4.56
Age group 13-18	5.84	5.31	7.25	7.95
Age group 19-25	7.33	8.60	9.08	10.33
Age group 26-45	22.52	22.96	24.74	21.52
Age group 46-65	26.47	25.24	22.46	22.69
Age group 66-85	19.61	20.34	15.16	18.29
Age group 86+	2.05	2.58	1.48	2.28
Male	43.17	42.26	42.39	43.46
Household income/100,000	3.55 (2.51)	3.43 (1.83)	3.59 (2.10)	3.50 (1.99)
Contact characteristics				
Contact type				
Simple encounter	6.07	5.62	2.31	2.48
Telephone contact	6.76	8.14	3.80	3.53
Consultation	86.61	85.83	93.69	93.74
Home visit	0.57	0.42	0.19	0.25
Diagnosis				
Upper RT symptoms and infections	30.08	26.64	28.86	28.50
Acute bronchitis	13.15	10.93	10.96	10.48
Chronic bronchitis/ COPD	6.14	6.43	3.20	3.80
Ear infection	8.45	9.75	10.39	9.70
Pneumonia	11.21	14.72	14.25	16.95
Sinusitis	13.33	15.31	12.48	13.51
Acute tonsilitis	10.06	10.13	14.24	14.07
Other respiratory diagnoses	6.57	6.07	5.58	3.85
Practice/list characteristics				
List length/100	11.49 (3.01)	10.04 (3.17)	11.89 (3.42)	9.60 (3.06)
Open list	14.36	18.46	17.04	15.20
Group practice	88.48	96.07	95.37	93.27
Proportion females	0.49	0.49	0.49 (0.09)	0.49 (0.08)
Average age	40.70 (5.33)	39.84 (5.87)	39.30 (5.43)	41.22 (6.07)
Proportion low income	0.19 (0.07)	0.20 (0.08)	0.19 (0.07)	0.19 (0.06)
Observations	13,965	5,270	30,286	10,562
Patients	10,233	4,173	27,268	9,660
GPs	154	154	496	496

Percentages are reported for the discrete variables; mean (standard deviation) – for continuous variables

Further, we estimate Eq. 1 for both outcomes using the datasets of regular GPs and locums who switched between remuneration systems. We estimate the models with GP fixed effects and present the results for the dataset, including all RTI contacts in the main text. The results on the datasets, including only the first (the only contacts) during RTI episodes, are presented in Additional file 1 available online.

We use a linear probability model with ordinary least squares (OLS) with standard errors clustered at the GP level for all the Models mentioned in the methods section. However, we also applied generalized linear models (logit and probit) to the same datasets, and the results (Tables S4-S7 in Additional file 1) align with the OLS model.

## Results

We have divided the results section into three parts. First, we present the results for all regular GPs on antibiotic prescribing and non-PcV antibiotics chosen when prescribing antibiotics. Second, we examine the same outcomes for the population of locums. Last, we examine within-subject effects for antibiotic prescribing and non-PcV antibiotics chosen when prescribing antibiotics for both regular GPs and locums that have switched remuneration schemes.

### Regular GPs

Table 6 presents the estimation results of Model 1 and Model 2 for regular GPs on the main datasets and the datasets, including only the first (or the only contact) during an RTI episode. Estimation results show that, given all other factors constant, the probability of antibiotic prescription during the first contact of RTI was 2% points higher for GPs paid by FFS and CAP than for salaried GPs. However, the difference becomes lower (1.4% points) if we include all contacts in the analysis. Moreover, GPs reimbursed by FFS and CAP were 4.5–4.6% points more likely to choose non-PcV drugs when prescribing an antibiotic, depending on the order of the contacts.

**Table 6:** Estimation results of the models 1 and 2 with linear probability model (OLS) for regular GPs

	Dependent variable:			
	Antibiotic is prescribed (Model 1)		Non-PcV is chosen (Model 2)	
	all RTI contacts	first (or the only) contacts for RTI episodes	all RTI contacts	first (or the only) contacts for RTI episodes
FFS+CAP	0.014^***^ (0.005)	0.020^***^ (0.006)	0.045^***^(0.013)	0.046^***^(0.015)
GP characteristics				
Specialist	-0.004^*^ (0.002)	-0.006^***^ (0.002)	-0.035^***^ (0.005)	-0.039^***^ (0.006)
Male	0.000 (0.002)	0.000 (0.003)	0.025^***^ (0.007)	0.025^***^ (0.008)
Age	0.001^***^ (0.000)	0.001^***^ (0.000)	0.002^***^ (0.000)	0.003^***^ (0.000)
Patient characteristics				
Age group 3-6	0.025^***^ (0.001)	0.023^***^ (0.001)	-0.028^***^ (0.003)	-0.021^***^ (0.003)
Age group 7-12	0.026^***^ (0.001)	0.021^***^ (0.001)	-0.092^***^ (0.005)	-0.103^***^ (0.005)
Age group 13-18	0.025^***^ (0.001)	0.025^***^ (0.001)	-0.104^***^ (0.006)	-0.121^***^ (0.006)
Age group 19-25	0.039^***^ (0.001)	0.046^***^ (0.001)	-0.086^***^ (0.005)	-0.093^***^ (0.006)
Age group 26-45	0.036^***^ (0.001)	0.046^***^ (0.001)	-0.039^***^ (0.005)	-0.047^***^ (0.006)
Age group 46-65	0.039^***^ (0.001)	0.054^***^ (0.001)	0.049^***^ (0.005)	0.049^***^ (0.006)
Age group 66-85	0.039^***^ (0.001)	0.057^***^ (0.001)	0.071^***^ (0.005)	0.074^***^ (0.006)
Age group 86+	0.021^***^ (0.001)	0.034^***^ (0.002)	0.007 (0.006)	0.023^***^ (0.007)
Male	-0.002^***^ (0.000)	-0.002^***^ (0.000)	-0.014^***^ (0.001)	-0.015^***^ (0.001)
Household income/100,000	0.0004^***^ (0.000)	0.0003^***^ (0.000)	0.001^***^ (0.000)	0.001^***^ (0.000)
Patient is listed with the GP	0.002 (0.003)	0.003 (0.003)	0.027^***^ (0.009)	0.021^**^ (0.009)
Contact characteristics				
Telephone contact	0.006^***^(0.001)	0.009^***^(0.001)	0.037^***^(0.003)	0.016^***^(0.005)
Consultation	0.141^***^(0.001)	0.156^***^(0.001)	-0.124^***^(0.003)	-0.107^***^(0.004)
Home visit	0.075^***^(0.003)	0.083^***^(0.004)	-0.087^***^(0.009)	-0.089^***^(0.012)
Diagnosis				
Upper RT symptoms and infections	-0.160^***^(0.003)	-0.178^***^(0.004)	-0.179^***^(0.005)	-0.195^***^(0.005)
Chronic bronchitis/COPD	-0.166^***^(0.003)	-0.199^***^(0.004)	0.131^***^(0.004)	0.155^***^(0.005)
Ear infection	0.100^***^(0.004)	0.112^***^(0.005)	-0.274^***^(0.006)	-0.288^***^(0.006)
Pneumonia	0.084^***^(0.004)	0.172^***^(0.005)	-0.068^***^(0.005)	-0.096^***^(0.006)
Sinusitis	0.139^***^(0.004)	0.164^***^(0.004)	-0.193^***^(0.006)	-0.199^***^(0.006)
Acute tonsilitis	0.297^***^(0.005)	0.346^***^(0.006)	-0.447^***^(0.005)	-0.455^***^(0.006)
Other respiratory diagnoses	-0.159^***^ (0.003)	-0.190^***^(0.004)	-0.044^***^(0.005)	-0.056^***^(0.006)
Practice/list characteristics				
List length/100	0.002^***^(0.000)	0.002^***^(0.000)	0.006^***^(0.001)	0.007^***^(0.001)
Open list	0.006^***^(0.001)	0.007^***^(0.002)	0.011^***^(0.004)	0.010^**^(0.004)
Group practice	-0.010^*^(0.005)	-0.016^***^(0.006)	-0.028^**^(0.013)	-0.028^*^(0.015)
Proportion females	0.017 (0.012)	0.023 (0.014)	0.037 (0.037)	0.031 (0.041)
Average age	0.001^***^(0.000)	0.001^***^(0.000)	-0.001 (0.001)	-0.001 (0.001)
Proportion low income	0.045^***^(0.016)	0.079^***^(0.019)	-0.208^***^(0.055)	-0.197^***^(0.058)
Month FE	Yes	Yes	Yes	Yes
Year FE	Yes	Yes	Yes	Yes
Municipality FE	Yes	Yes	Yes	Yes
Observations	9,977,798	6,972,642	1,487,309	1,162,589
*R* ^2^	0.167	0.208	0.166	0.167

Robust standard errors clustered at the GP level are shown in parentheses. Estimation results for year, month and municipality dummies are omitted to save space and are available upon request

*p<0.1; **p<0.05; ***p<0.01, *FE F*ixed effects

The above-mentioned results can be interpreted in relative terms in the following way. On average, in 11.95% of all RTI contacts (and in 13.31% of first contacts), salaried GPs prescribed an antibiotic treatment. According to the model prediction, the corresponding rates for the GPs paid by FFS and CAP will be 13.35% and 15.31%, respectively. Thus, in relative terms, the probability of antibiotic prescription by a GP paid by FFS and CAP is 12–15% higher than for a salaried GP. Regarding the choice of antibiotic, the relative difference in the probability of prescribing non-PcV between salaried GPs and those reimbursed by FFS and CAP is 9% and 11% for all contacts and the first contacts, respectively. The results correspond to the estimations of logit and probit regressions (Tables S4-S5 in Additional file 1), which indicate a 8–21% difference in odds of antibiotic prescription and a 15–26% difference in odds of choosing non-PcV.

Being a specialist, according to our model, did not have major or significant effect on the probability of an antibiotic prescription, but it was associated with 3.5–3.9% points lower probability of choosing non-PcV. Male GPs were more likely to prescribe non-PcV. The probability of antibiotic prescription and choice of non-PcV antibiotic increased with the GP age. We also found significant effects of patient characteristics on the probability of antibiotic prescription and of choosing non-PcV. According to the estimation results, both probabilities were higher for female than for male patients, and for patients with higher household income. Patients listed with the GP had higher probability of getting a non-PcV antibiotic. The probability of antibiotic prescription was the lowest for kids from 0 to 2, and highest for patients of age 66–85. The lowest probability of getting non-PcV was for the age group 13–18 and the highest for patients in the age group 66–85.

Antibiotics were more likely to be prescribed during consultation at the GP office (in contrast to telephone contact or home visit) and when the GP used the diagnosis code for acute tonsilitis. When an antibiotic has been prescribed, GPs were more likely to choose non-PcV drugs during telephone contact and for the diagnosis of chronic bronchitis/COPD. According to the model, practice characteristics had significant effects on the outcome variables. GPs with longer and open lists were more likely to prescribe an antibiotic for RTI and to choose a non-PcV antibiotic. Proportion of patients with low income was positively associated with the probability of antibiotic prescription and negatively associated with the probability of choosing a broad-spectrum antibiotic. GPs with older patients on the list were more likely to prescribe antibiotics.

### Locums

Table 7 presents the estimation results of Model 1 and Model 2 for locums on the main datasets and the datasets, including only the first (or the only) contacts during RTI episodes. Estimation results show that, given all other factors constant, the probability of antibiotic prescription did not significantly differ between FFS and salaried GPs. Results also show that locums reimbursed by FFS were 2.6 and 3.2% points more likely to choose non-PcV drugs when prescribing an antibiotic during the first contact and generally, respectively. In relative terms, the probability of non-PcV prescription by a GP paid by FFS is 7% higher than for a salaried GP. The results correspond to the results of logit and probit regressions (Tables S6-S7 in Additional file 1).

**Table 7:** Estimation results of the models 1 and 2 with linear probability model (OLS) for locum GPs

	Dependent variable:			
	Antibiotic is prescribed (Model 1)		Non-PcV is chosen (Model 2)	
	all RTI contacts	first (or the only) contacts for RTI episodes	all RTI contacts	first (or the only) contacts for RTI episodes
FFS	0.001 (0.004)	0.004 (0.004)	0.032^***^(0.011)	0.026^**^(0.011)
GP characteristics				
Specialist	-0.009^**^(0.005)	0.011^**^(0.005)	-0.025^**^(0.012)	-0.024^*^(0.013)
Male	-0.002 (0.002)	-0.002 (0.003)	0.013 (0.009)	0.012 (0.009)
Age	0.001^***^(0.000)	0.002^***^(0.000)	0.004^***^(0.000)	0.005^***^(0.000)
Patient characteristics				
Age group 3-6	0.025^***^(0.002)	0.023^***^ (0.002)	-0.014^***^(0.006)	-0.011 (0.006)
Age group 7-12	0.017^***^(0.002)	0.011^***^ (0.002)	-0.047^***^(0.008)	-0.056^***^(0.008)
Age group 13-18	0.014^***^(0.002)	0.014^***^(0.002)	-0.051^***^(0.008)	-0.067^***^(0.008)
Age group 19-25	0.024^***^(0.002)	0.028^***^(0.002)	-0.032^***^(0.008)	-0.040^***^(0.009)
Age group 26-45	0.021^***^(0.002)	0.027^***^ (0.002)	0.009 (0.008)	0.000 (0.008)
Age group 46-65	0.026^***^(0.002)	0.035^***^ (0.002)	0.102^***^(0.008)	0.100^***^(0.008)
Age group 66-85	0.028^***^(0.002)	0.041^***^(0.002)	0.136^***^(0.009)	0.136^***^(0.009)
Age group 86+	0.000 (0.002)	0.009^***^ (0.003)	0.067^***^(0.012)	0.086^***^(0.013)
Male	-0.004^***^(0.001)	-0.004^***^ (0.001)	-0.019^***^(0.002)	-0.018^***^(0.002)
Household income/100,000	0.0003^***^ (0.000)	0.0002^***^ (0.000)	0.001^***^ (0.000)	0.001^***^ (0.000)
Contact characteristics				
Telephone contact	0.018^***^(0.002)	0.012^***^ (0.002)	0.077^***^(0.010)	0.069^***^(0.016)
Consultation	0.130^***^(0.002)	0.145^***^ (0.002)	-0.169^***^(0.008)	-0.142^***^(0.013)
Home visit	0.062^***^(0.007)	0.049^***^ (0.009)	-0.118^***^(0.022)	-0.109^***^(0.028)
Diagnosis				
Upper RT symptomsand infections	-0.127^***^(0.005)	-0.142^***^ (0.006)	-0.172^***^(0.008)	-0.185^***^(0.009)
Chronic bronchitis/COPD	-0.114^***^(0.005)	-0.122^***^ (0.006)	0.207^***^(0.009)	0.231^***^(0.010)
Ear infection	0.190^***^(0.006)	0.217^***^ (0.007)	-0.276^***^(0.009)	-0.285^***^(0.009)
Pneumonia	0.184^***^(0.006)	0.277^***^ (0.007)	-0.067^***^(0.008)	-0.099^***^(0.010)
Sinusitis	0.144^***^(0.006)	0.160^***^ (0.007)	-0.216^***^(0.011)	-0.216^***^(0.013)
Acute tonsilitis	0.390^***^ (0.006)	0.446^***^ (0.007)	-0.394^***^(0.008)	-0.393^***^(0.009)
Other respiratorydiagnoses	-0.124^***^(0.005)	-0.144^***^ (0.006)	-0.027^***^(0.009)	-0.047^***^(0.011)
Practice/list characteristics				
List length/100	0.001^***^(0.000)	0.001^**^ (0.000)	0.002 (0.001)	0.002 (0.001)
Open list	0.005^***^(0.002)	0.004^**^ (0.002)	0.004 (0.003)	0.006 (0.006)
Group practice	-0.003 (0.006)	-0.009 (0.008)	-0.032 (0.020)	-0.038^*^(0.022)
Proportion females	-0.005 (0.008)	-0.007 (0.009)	-0.017 (0.031)	-0.002 (0.030)
Average age	0.001^***^(0.000)	0.001^***^ (0.000)	0.001 (0.001)	0.001 (0.001)
Proportion low income	0.009 (0.017)	0.031^**^(0.019)	-0.083 (0.062)	-0.054 (0.062)
Month FE	Yes	Yes	Yes	Yes
Year FE	Yes	Yes	Yes	Yes
Municipality FE	Yes	Yes	Yes	Yes
Observations	1,520,168	1,164,473	234,614	193,672
*R* ^2^	0.183	0.223	0.177	0.171

Robust standard errors clustered at the GP level are shown in parentheses. Estimation results for year, month and municipality dummies are omitted to save space and are available upon request

*p<0.1; **p<0.05; ***p<0.01, *FE* Fixed effects

According to our model, specialists and younger locums were less likely to give an antibiotic prescription and to choose non-PcV antibiotics. Male GPs did not significantly differ from females in terms of the probability of antibiotic prescription and choice of antibiotic. Both probabilities were higher for female patients than for male. The probability of antibiotic prescription and the probability of getting non-PcV was the highest for patients from the age group 66–85.

Similarly to regular GPs, locums prescribed antibiotics with the highest probability for acute tonsilitis and prescribed non-PcV for chronic bronchitis/COPD. The probability of receiving an antibiotic prescription and a non-PcV antibiotic was higher during a consultation at the GP office and during a telephone consultation, respectively. Models show some significant but mostly minor effects of practice characteristics on the outcome variables. Locums working on longer and open lists, and with older patients on the list, were slightly more likely to prescribe an antibiotic for RTI. Higher proportion of patients with low income was associated with higher probability of antibiotic prescription during the first contact.

### GPs switching remuneration scheme

In Table 8, we present estimation results for GPs switching remuneration schemes. These results are based on the datasets which include all RTI contacts. The results for the datasets, including the first (or the only) contacts, are similar and are available in in Additional file 1 (Table S8). Estimation results by logit and probit models are consistent with OLS and are available upon request. We find that, when working under FFS and CAP, regular GPs were more likely to prescribe an antibiotic for RTI and to prescribe a non-PcV antibiotic than when being salaried. The difference in probabilities of antibiotic prescription and choosing non-PcV is 1.6 and 4.5% points, respectively, which corresponds to a 14% and 8% difference in relative terms. However, we did not find any significant difference in the behaviour of locums depending on whether they worked under FFS or were salaried. Further, we did not find any significant difference in the GPs’ behaviour depending on whether they were a non-specialist or became specialists later on. We found that female patients were slightly more likely to get an antibiotic and to get a broader-spectrum antibiotic, but only from locum GPs. The effect of patient age and the diagnosis on the outcomes have a similar pattern as in the previous models, except that we didn’t find any significant difference from the reference group in the choice of antibiotic prescribed to the patients from age group 3–25 by regular GPs and to the patients from age group 19–45 by locums. According to the models, practice or list characteristics have little or no effect on prescription behaviour, except that higher proportion of patients with low income was associated with lower probability of antibiotic prescription by locums, and that locums were slightly more likely to prescribe an antibiotic when working with longer and more open lists. 

**Table 8:** Estimation results for GPs switching the remuneration type with linear probability model (OLS) on the dataset including all RTI contacts

	Dependent variable:			
	Antibiotic is prescribed		Non-PcV is chosen	
	Regular GPs	Locums	Regular GPs	Locums
FFS/FFS+CAP	0.016^***^(0.005)	0.001 (0.004)	0.045^**^(0.017)	0.001 (0.009)
GP characteristics				
Specialist	-0.005 (0.005)	0.004 (0.010)	-0.009 (0.016)	-0.036^*^(0.022)
Patient characteristics				
Age group 3-6	0.014^**^(0.006)	0.031^***^(0.004)	0.000 (0.020)	-0.029^**^(0.013)
Age group 7-12	0.028^***^(0.007)	0.018^***^(0.005)	-0.032 (0.023)	-0.040^**^(0.116)
Age group 13-18	0.024^***^(0.008)	0.023^***^(0.003)	-0.019 (0.027)	-0.049^**^(0.019)
Age group 19-25	0.038^***^(0.008)	0.029^***^(0.005)	-0.011 (0.025)	-0.037^*^(0.019)
Age group 26-45	0.040^***^(0.008)	0.028^***^(0.005)	0.054^**^(0.025)	0.003 (0.017)
Age group 46-65	0.037^***^(0.008)	0.033^***^(0.005)	0.136^***^(0.023)	0.096^***^(0.018)
Age group 66-85	0.029^***^(0.007)	0.033^***^(0.005)	0.144^***^(0.025)	0.134^***^(0.017)
Age group 86+	0.003 (0.005)	0.004 (0.006)	0.133^***^(0.036)	0.100^***^(0.025)
Male	-0.001 (0.002)	-0.005^***^(0.002)	-0.009 (0.007)	-0.026^***^(0.005)
Household income	0.001^**^ (0.000)	0.000 (0.000)	0.000 (0.002)	0.003^***^ (0.001)
Contact characteristics				
Telephone contact	0.010 (0.007)	0.025^***^(0.004)	-0.027 (0.019)	0.122^***^(0.020)
Consultation	0.126^***^(0.010)	0.142^***^(0.005)	-0.131^***^(0.017)	-0.140^***^(0.015)
Home visit	0.059^***^(0.016)	0.056^***^(0.013)	-0.010 (0.042)	-0.118^**^(0.056)
Diagnosis				
Upper RT symptomsand infections	-0.146^***^(0.017)	-0.165^***^(0.015)	-0.141^***^(0.016)	-0.135^***^(0.021)
Chronic bronchitis/COPD	-0.144^***^(0.018)	-0.140^***^(0.016)	0.130^***^(0.025)	0.251^***^(0.023)
Ear infection	0.089^***^(0.019)	0.162^***^(0.018)	-0.257^***^(0.027)	-0.248^***^(0.021)
Pneumonia	0.052^***^(0.018)	0.160^***^(0.017)	-0.037^*^(0.021)	-0.006 (0.024)
Sinusitis	0.106^***^(0.017)	0.134^***^(0.014)	-0.175^***^(0.028)	-0.151^***^(0.038)
Acute tonsilitis	0.258^***^(0.025)	0.345^***^(0.017)	-0.379^***^(0.024)	-0.323^***^(0.020)
Other respiratorydiagnoses	-0.152^***^(0.018)	-0.159^***^(0.016)	-0.019 (0.020)	0.015 (0.023)
Practice/list characteristics				
List length/100	0.000 (0.002)	0.001^***^(0.000)	0.002 (0.007)	0.000 (0.001)
Open list	-0.003 (0.004)	0.007^***^(0.003)	-0.007 (0.016)	0.006 (0.007)
Group practice	0.015^*^(0.008)	0.003 (0.007)	0.017 (0.034)	0.030^*^(0.016)
Proportion females	0.059 (0.056)	-0.005 (0.012)	0.180 (0.279)	-0.018 (0.034)
Average age	-0.001 (0.001)	0.000 (0.000)	-0.003 (0.006)	0.000 (0.001)
Proportion low income	-0.063 (0.074)	-0.087^***^(0.019)	0.082 (0.273)	-0.044 (0.054)
Month FE	Yes	Yes	Yes	Yes
Year FE	Yes	Yes	Yes	Yes
Municipality FE	Yes	Yes	Yes	Yes
GP FE	Yes	Yes	Yes	Yes
Observations	156,326	251,783	19,235	40,848
*R*2	0.165	0.221	0.264	0.257

Robust standard errors clustered at the GP level are shown in parentheses. Estimation results for year, month and municipality dummies are omitted to save space and are available upon request. GP gender and GP age are omitted due to no or limited within-GP variation

*p<0.1; **p<0.05; ***p<0.01, *FE F*ixed effects

## Discussion and conclusions

The increased use of antibiotics is the leading cause of the growing rates of AR. This has led to antibiotics becoming less effective in treating infectious diseases. Therefore, it is essential to investigate factors that may result in antibiotic overprescription. Our findings support the hypothesis that the GP remuneration scheme may be one of those factors.

We find that regular GPs paid by a mix of FFS and CAP prescribed antibiotics for RTIs more often than salaried GPs. Moreover, salaried GPs were more likely to choose narrow-spectrum PcV when prescribing an antibiotic. More specifically, we found that the difference in the probability of antibiotic prescription between different remuneration schemes was slightly larger when we analysed the dataset including only the first (or the only) consultations of RTI episodes. This result may be explained by the fact that patients of the salaried GPs who had not received an antibiotic during their first contact were more likely to return for a prescription later, which may be a sign of sometimes unnecessarily applied strategy of watchful waiting by salaried GPs. Another possible explanation for the above-mentioned result is that the GPs paid by FFS and CAP invite healthy patients for control visits more often. However, the effect of FFS and CAP on the outcomes remains positive when all consultations are included in the analysis. It is theoretically impossible with our data to identify whether salaried GPs under-prescribed antibiotics or whether GPs paid by FFS and CAP provided unnecessary prescriptions. However, if we assume that patients return for antibiotic treatment if they do not get better, our results indicate that the GPs paid by FFS and CAP overprescribed antibiotics. However, further analysis is preferably based on the data on prescriptions from PCECs and emergency hospital admissions for infectious diseases. Finally, our results may be explained by some other systematic differences between GPs choosing to work under a specific remuneration scheme, e.g. patient selection, or differences in GPs’ profit-maximizing preferences. We attempted to provide additional insight into this and analysed GPs who switched remuneration schemes controlling for the GP-fixed effects, in other words, GPs’ unobserved characteristics. The analysis shows that the differences in antibiotic prescription remain significant. It is questionable whether the sample of GPs switching remuneration schemes is representative of the general GP population. However, we found no systematic differences between those groups except the gender of the GPs. Moreover, we did not find any pattern in whether GPs systematically switched from one system to another.

The analysis of locum GPs does not show any difference in the probability of antibiotic prescription between locums paid by FFS and salaried locums. Therefore, we do not find signs of antibiotic overprescription by the locums paid by FFS. When analysing the choice of antibiotic, we found that FFS locums were more likely to choose non-PcV during the first contact but only according to the linear model. When including all contacts in the analysis, the difference between locums paid by FFS and salaried locums becomes larger and statistically significant for all models. This result may indicate that FFS locums chose the wrong antibiotic treatment more often. This could happen if the locums paid by FFS, during the first contacts, chose PcV when another antibiotic was needed and vice versa, or if a non-PcV antibiotic was chosen incorrectly. Therefore, they might have to prescribe another broad-spectrum antibiotic during the next visit.

When analysing locums who switched remuneration types and controlling for unobserved GP-related factors, we no longer find any significant difference in antibiotic prescription behaviour between salaried locums and those paid by FFS. Thus, we did not find any effect of FFS on antibiotic prescription rates for RTIs among locum GPs. However, we found that a mix of FFS and CAP may contribute to higher antibiotic prescription rates. These results may occur if the CAP component in the remuneration scheme purely explains the effect of the FFS and CAP mix. The lack of CAP payments together with the instability of temporary contracts may weaken locums’ incentives to prescribe antibiotics for patient retention purposes. Brekke et al. [14] argued that locums are highly representative of the general population of Norwegian GPs since many locums further get regular contracts. However, regular GPs and locums may also differ in their characteristics and behaviour. For example, locums working under FFS may be more experienced or less risk-averse in antibiotic prescription behaviour than salaried locums. At the same time, this difference may be smaller for regular GPs. It is also important to notice the strict attitudes towards antibiotic use in Norway and the relatively low levels of AR [47]. Moreover, some of the salaried GPs (both regular GPs and locums) had bonus agreements with the municipalities and received a part of the FFS payments in addition to a fixed salary. Therefore, the effects of FFS and CAP, and pure FFS may be underestimated and may be stronger in another institutional or geographical setting.

One limitation of our study is that the GPs’ use of diagnoses may be related to their likelihood of prescribing antibiotics. Patients may present several symptoms during a single consultation, and all symptoms may not be recorded by the GP. If GPs are more likely to use a respiratory tract infection diagnosis when they decide to prescribe antibiotics, this may dilute our results, possibly underestimating differences between remuneration schemes. Also, by exclusively studying antibiotic use for respiratory tract infections, we may be looking at the most clear-cut clinical cases. Much of the variation in antibiotics use, and perhaps much of the unnecessary prescriptions, may relate to edge cases where a respiratory tract infection is less certain. Another important limitation is that we are unable to draw a conclusion about the appropriateness of the antibiotic prescriptions made by the GPs working under different remuneration schemes.

However, if a sample of regular GPs switching remuneration schemes is representative and if their medical decision-making is not affected by other factors than we controlled for, including remuneration type, patient characteristics, and GP specialization, and if it is unlikely that all GPs underprescribe antibiotics, the results may indicate a need for policy solutions. Whether the proportion of salaried contracts should be increased remains an open question. To make informed policy decisions, it is important to understand the effect of remuneration type on other aspects of healthcare quality and health outcomes, e.g. access equity, waiting times, treatment effectiveness, cost-efficiency, and the delivery of preventive care. As mixed payment models are generally considered to increase productivity, more nuanced policy interventions may be needed. Such policies may be differentiated depending on the remuneration scheme of the GP and may include better monitoring of the prescriptions or adjustments to the remuneration scheme to influence prescription practice, e.g. antibiotic-related pay-for-performance indicators. 

## Supplementary Information

Supplementary Material 1.

## Data Availability

Due to the sensitive nature of the data, which are derived from nationwide Norwegian health registries including the Control and Payment of Health Reimbursements Database (KUHR), the National GP Registry (Fastlegeregisteret), the Norwegian Prescribed Drug Registry (NorPD), and Statistics Norway (SSB), we are legally prohibited from sharing individual-level data publicly. Access to these data requires specific approval from the relevant Norwegian data protection authorities and can only be granted for research conducted under strict confidentiality and security protocols.

## Footnotes

1 Our data does not contain information about special agreements. Therefore, the difference in antibiotic rates between salaried GPs and GPs paid by a mix of FFS and CAP may be underestimated.

2 In larger municipalities, PCECs may also be available during the daytime.

3 In the case of multiple diagnoses, we chose the most relevant for prescribing an antibiotic. This choice was based on the share of contacts with a specific diagnosis that resulted in an antibiotic prescription (See Table S1 in Additional file 1).

4 As in the previous literature on RTI episodes, we marked contacts that happened within the next 30 days after the previous one as re-consultations or control contacts for the same RTI episode, where the episode start date was defined as the RTI contact date, given that no other RTI contact has been registered within the previous 30 days.

5 In about 1% of all matched observations, more than one contact for RTI fulfilled the above-mentioned criteria. Therefore, we assigned the prescription to the contact closest to the antibiotic collection date. In about 1% of all antibiotic prescriptions, more than one antibiotic drug prescribed by a GP was collected by a patient simultaneously (on the same day). We treat such prescriptions as antibiotic prescriptions in combination.

6 We consider antibiotics prescribed in combination as one prescription or as a fact of receiving an antibiotic treatment.

7 We treat prescriptions of antibiotics in combination as separate observations.

8 After-tax income per consumption unit, EU scale in NOK. Total household taxable and non-taxable income, minus taxes, divided on the number of consumption units in the household. The number of consumption units is calculated by using the ‘modified’ OECD scale or the EU scale, where the first adult is given a value of 1, any additional adult is given the value of 0.5, and each child is given a value of 0.3. The number of consumption units in a household consisting of two adults and two children is thus 2.1, according to this method.

9 Percentage of patient with income within the first two percentiles, measured for adults above the age of 20.

10 Due to the data protection issues, we had access to anonymized municipality ID. During the study period, some Norwegian municipalities have been restructured and recoded, which also affected the anonymized IDs. Therefore, we had to make the following adjustments. We assume that most of the contracts remain geographically in the same location. If the municipality code has been changed throughout time, but no contracts previously located in other municipalities receive the same new code, as well as no contracts from the old municipality appear to be in a new municipality different from the municipality of other contracts, we assume that the municipality remains the same but is just recoded. In case several contracts from different municipalities receive a new code, we treat this as municipalities merging. Merged municipalities are treated differently from the initial ones. Suppose several contracts from a current municipality receive a new code, which is the same as the existing code for another municipality. In that case, we further assign the existing code of the new municipality to the current one. We found no cases where municipalities were divided into several new municipalities.

## Abbreviations

AR: Antibiotic resistance
ATC: Anatomical therapeutic chemical classification system
CAP: Capitation
FFS: Fee–for–service
GP: General practitioner
ICPC: International Classification of Primary Care
KUHR: Control and Payment of Health Reimbursements Database
NorPD: Norwegian Prescribed Drug Registry
OECD: Organisation for Economic Co–operation and Development
PcV: Phenoxymethylpenicillin
RTI: Respiratory tract infection
SSB: Statistics Norway
