# Supplementary material for: Can the remuneration scheme of general practitioners affect their antibiotic prescription behaviour?
Source: Health Econ Rev. 2026 Feb 6;16:29. doi: 10.1186/s13561-026-00736-w (PMC12977734; doi:10.1186/s13561-026-00736-w)
Supplement: Supplementary file 1 — Supplementary Material 1. [file 13561_2026_736_MOESM1_ESM.pdf]

Supplementary Materials

Table S1 Diagnoses ranks

ICPC-2 code	Diagnosis	Number of contacts	Share of contacts leading to antibiotic prescription	Rank
R72	Strep throat	149671	0.620	1
R76	Tonsillitis acute	149197	0.451	2
H71	Acute otitis media/myringitis	286576	0.414	3
R75	Sinusitis acute/chronic	548101	0.346	4
R81	Pneumonia	491143	0.311	5
R71	Whooping cough	15651	0.298	6
R78	Acute bronchitis/bronchiolitis	699794	0.210	7
R73	Boil/abscess nose	5375	0.156	8
H72	Serous otitis media	72273	0.153	9
R79	Chronic bronchitis	9188	0.151	10
R09	Sinus symptom/complaint	172084	0.128	11
H74	Chronic otitis media	12916	0.125	12
R83	Respiratory infection other	601693	0.117	13
R74	Upper respiratory infection acute	2722660	0.085	14
R21	Throat symptom/complaint	576073	0.078	15
R90	Hypertrophy tonsils/adenoids	58626	0.071	16
R05	Cough	1418399	0.059	17
R29	Respiratory symptom/complaint other	107912	0.059	18
R95	Chronic obstructive pulmonary dis	1308361	0.054	19
R89	Congenital anomaly respiratory	3537	0.051	20
R77	Laryngitis/tracheitis acute	41936	0.046	21
R80	Influenza	572548	0.041	22
R28	Limited function/disability (r)	3041	0.036	23
R82	Pleurisy/pleural effusion	14338	0.034	24
R25	Sputum/phlegm abnormal	3420	0.031	25
R24	Haemoptysis	24651	0.027	26
R08	Nose symptom/complaint other	69322	0.026	27
R01	Pain respiratory system	9111	0.024	28
R02	Shortness of breath/dyspnoea	686514	0.020	29
R23	Voice symptom/complaint	78211	0.019	30
R03	Wheezing	3731	0.018	31
R84	Malignant neoplasm bronchus/lung	230184	0.018	32
R26	Fear of cancer respiratory system	2825	0.017	33
R92	Neoplasm respiratory unspecified	6241	0.017	34
R85	Malignant neoplasm respiratory, other	29585	0.017	35
R07	Sneezing/nasal congestion	200894	0.015	36
R27	Fear of respiratory disease, other	670709	0.015	37
R99	Respiratory disease other	1740685	0.013	38
R88	Injury respiratory other	9534	0.011	39
R04	Breathing problem, other	121677	0.011	40
R86	Benign neoplasm respiratory	4043	0.009	41
R06	Nosebleed/epistaxis	130009	0.009	42
R87	Foreign body nose/larynx/bronchi	6743	0.007	43
R98	Hyperventilation syndrome	4377	0.006	44

Table S2 Descriptive statistics for the datasets including the first or the only contact for each RTI episode, decomposed by the remuneration types (Model 1)

	Regular GPs		Locums	
	FFS+CAP	Salary	FFS	Salary
Dependent variable				
Antibiotic prescriptions for RTI	16.69	13.31	16.40	16.73
GP characteristics				
Specialist	66.93	35.75	8.89	6.32
Male	64.68	52.66	48.73	60.43
Age	49.97 [illegible]	44.55 (11.32)	37.59 (10.16)	40.14 (13.72)
Patient characteristics				
Age group 0-2	7.08	6.97	8.53	7.54
Age group 3-6	6.22	5.58	6.89	6.07
Age group 7-12	4.70	4.02	4.85	4.46
Age group 13-18	7.54	6.70	7.97	7.84
Age group 19-25	8.16	8.76	9.11	9.24
Age group 26-45	22.57	19.09	24.59	19.63
Age group 46-65	24.09	23.03	21.67	22.15
Age group 66-85	17.65	22.52	14.68	20.13
Age group 86+	1.99	3.33	1.71	2.93
Male	44.26	44.21	42.87	44.94
Household income/100,000	3.68 (3.57)	3.40 (1.99)	3.66 (4.20)	3.43 (7.93)
Patient is listed with the GP	77.46	63.05	0.03	0.02
Contact characteristics				
Contact type				
Simple encounter	13.96	14.28	9.36	8.33
Telephone contact	8.96	10.30	6.10	6.64
Consultation	76.86	75.01	84.36	84.66
Home visit	0.22	0.40	0.18	0.37
Diagnosis				
Upper RT symptoms and infections	53.48	53.16	58.62	56.74
Acute bronchitis	7.63	6.58	6.51	6.59
Chronic bronchitis/ COPD	7.86	10.70	5.43	7.50
Ear infection	4.07	3.99	4.18	4.23
Pneumonia	3.72	5.22	3.93	5.85
Sinusitis	5.50	4.89	5.31	5.11
Acute tonsilitis	3.45	3.29	3.82	4.10
Other respiratory diagnoses	14.28	12.17	12.19	9.88
Practice/list characteristics				
List length/100	13.02 (3.45)	8.66 (2.85)	12.54 (3.36)	9.21 (2.95)
Open list	19.94	15.47	16.76	14.42
Group practice	93.81	93.85	95.18	93.02
Proportion females	0.49 (0.10)	0.49 (0.10)	0.51 (0.10)	0.49 (0.09)
Average age	39.97 (5.39)	40.15 (6.55)	38.98 (5.62)	41.05 (6.15)
Proportion low income	0.18 (0.07)	0.21 (0.08)	0.18 (0.07)	0.20 (0.06)
Observations	6,640,644	331,998	1,044,036	120,437
Patients	2,554,971	177,554	703,961	91,062
GPs	5,217	1,020	3,575	1,412

Notes: Percentages are reported for the discrete variables; mean (standard deviation) – for continuous variables.

Table S3 Descriptive statistics for the datasets including the first or the only contact for each RTI episode leading to antibiotic prescriptions and decomposed by the remuneration types (Model 2)

	Regular GPs		Locums	
	FFS+CAP	Salary	FFS	Salary
Dependent variable				
Non-PcV antibiotic	51.21	43.68	41.20	39.43
GP characteristics				
Specialist	66.16	35.18	9.69	6.54
Male	65.73	55.44	50.01	62.57
Age	50.56 (10.76)	44.84 (11.22)	38.69 (11.09)	42.07 (14.39)
Patient characteristics				
Age group 0-2	6.11	6.46	7.62	6.11
Age group 3-6	7.11	6.81	8.48	7.20
Age group 7-12	4.98	4.79	5.37	4.71
Age group 13-18	6.60	6.36	6.96	7.64
Age group 19-25	8.82	10.23	9.56	10.05
Age group 26-45	24.94	22.27	25.75	21.64
Age group 46-65	24.13	22.45	21.08	22.48
Age group 66-85	15.72	18.27	13.78	17.99
Age group 86+	1.59	2.36	1.41	2.18
Male	42.93	42.25	41.87	43.83
Household income/100,000	3.71 (3.57)	3.49 (2.16)	3.68 (2.85)	3.51 (2.01)
Patient is listed with the GP	71.65	52.28	0.03%	0.04%
Contact characteristics				
Contact type				
Simple encounter	3.03	3.49	1.64	1.63
Telephone contact	2.18	3.35	1.51	1.66
Consultation	94.56	92.83	96.67	96.43
Home visit	0.23	0.33	0.18	0.28
Diagnosis				
Upper RT symptoms and infections	28.21	25.21	30.17	26.43%
Acute bronchitis	13.50	10.42	9.24	9.88%
Chronic bronchitis/ COPD	2.94	4.64	2.39	3.45%
Ear infection	9.52	10.96	11.40	10.93%
Pneumonia	10.29	14.75	12.28	16.97%
Sinusitis	14.86	14.14	12.57	12.94%
Acute tonsilitis	13.19	14.46	16.03	15.74%
Other respiratory diagnoses	7.49	5.43	5.92	3.66%
Practice/list characteristics				
List length/100	13.19 (3.49)	8.82 (2.97)	12.57 (3.36)	9.45 (2.96)
Open list	20.87	16.89	17.56	15.31
Group practice	92.77	94.75	95.14	94.31
Proportion females	0.49 (0.10)	0.49 (0.09)	0.50 (0.10)	0.49 (0.08)
Average age	40.06 (5.34)	39.60 (6.41)	39.08 (5.59)	41.05 (5.94)
Proportion low income	0.18 (0.08)	0.21 (0.08)	0.18 (0.07)	0.19 (0.06)
Observations	1,117,920	44,669	173,248	20,424
Patients	904,738	41,293	174,813	20,515
GPs	5,192	967	3,258	1,205

Notes: Percentages are reported for the discrete variables; mean (standard deviation) – for continuous variables.

Logit and probit regressions

Further, we present estimation results of the logit and probit regression of the main models with the R-package 'feglm'. This technique is based on the work of Gaure (2013)[1] and Stammann[2] et. al. (2016) and is suitable for big dataset that are infeasible to estimate otherwise due to memory limitations.

Tables S4-S5 present the estimation results of Models 1 and 2 with logit and probit regressions for regular GPs, while Tables S6-S7 present estimation results for locums.

According to Table S4, regular GPs paid by FFS and CAP were 21% more likely (odds ratio is equal to exp(0.193)≈1.21) to prescribe an antibiotic for RTI during the first/only contact of the infection episode than salaried regular GPs and were 16% more likely (odds ratio is equal to exp(0.146)≈1.16) to prescribe an antibiotic for RTI for all contacts than salaried regular GPs. Further, regular GPs paid by FFS and CAP were 26% more likely to choose a non-PcV antibiotic during the first/only contact (odds ratio is equal to exp(0.232)≈1.26) and for all contacts (odds ratio is equal to exp(0.233)≈1.26), compared to salaried regular GPs.

Table S4 Estimation results of the Models 1 and 2 with logit regression for regular GPs, log odds ratios.

	Dependent variable:			
	Antibiotic prescribed (Model 1)		Non-PcV chosen (Model 2)	
	all RTI contacts	first (the only) contacts for RTI episodes	all RTI contacts	first (the only) contacts for RTI episodes
FFS+CAP	0.146***	0.193***	0.233***	0.232***
	(0.052)	(0.058)	(0.064)	(0.073)
GP characteristics				
Specialist	-0.034*	-0.056***	-0.171***	-0.188***
	(0.018)	(0.020)	(0.028)	(0.030)
Male	0.004	0.005	0.123***	0.119***
	(0.023)	(0.025)	(0.035)	(0.038)
Age	0.006***	0.008***	0.011***	0.013***
	(0.001)	(0.001)	(0.001)	(0.001)
Patient characteristics				
Age group 3-6	0.211***	0.202***	-0.122***	-0.091***

[1] Gaure, S. (2013). "OLS with Multiple High Dimensional Category Variables". Computational Statistics and Data Analysis, 66.

[2] Stammann, A., F. Heiss, and D. McFadden (2016). "Estimating Fixed Effects Logit Models with Large Panel Data". Working paper.

	(0.007)	(0.008)	(0.014)	(0.015)
Age group 7-12	0.224***	0.180***	-0.432***	-0.495***
	(0.009)	(0.010)	(0.021)	(0.022)
Age group 13-18	0.198***	0.201***	-0.487***	-0.583***
	(0.010)	(0.011)	(0.025)	(0.028)
Age group 19-25	0.335***	0.404***	-0.396***	-0.437***
	(0.010)	(0.011)	(0.025)	(0.027)
Age group 26-45	0.303***	0.405***	-0.182***	-0.220***
	(0.010)	(0.010)	(0.023)	(0.026)
Age group 46-65	0.318***	0.469***	0.214***	0.202***
	(0.011)	(0.012)	(0.024)	(0.026)
Age group 66-85	0.309***	0.498***	0.332***	0.321***
	(0.011)	(0.012)	(0.025)	(0.027)
Age group 86+	0.091***	0.267***	-0.002	0.072**
	(0.015)	(0.017)	(0.030)	(0.033)
Male	-0.021***	-0.023***	-0.069***	-0.071***
	(0.003)	(0.003)	(0.005)	(0.005)
Household income/100,000	0.004***	0.002***	0.005***	0.005***
	(0.001)	(0.001)	(0.001)	(0.001)
Patient is listed with the GP	0.018	0.022	0.129***	0.102**
	(0.027)	(0.029)	(0.043)	(0.045)
Contact characteristics				
Telephone contact	0.106***	0.161***	0.209***	0.082***
	(0.019)	(0.026)	(0.019)	(0.029)
Consultation	1.773***	2.100***	-0.630***	-0.534***
	(0.016)	(0.020)	(0.016)	(0.021)
Home visit	1.243***	1.500***	-0.455***	-0.447***
	(0.034)	(0.041)	(0.051)	(0.059)
Diagnosis				
Upper RT symptoms and infections	-1.293***	-1.375***	-0.833***	-0.879***
	(0.020)	(0.023)	(0.024)	(0.026)
Chronic bronchitis/ COPD	-1.505***	-1.647***	1.247***	1.346***
	(0.022)	(0.026)	(0.033)	(0.036)
Ear infection	0.494***	0.602***	-1.224***	-1.275***
	(0.022)	(0.025)	(0.027)	(0.029)
Pneumonia	0.528***	0.901***	-0.352***	-0.461***
	(0.021)	(0.024)	(0.025)	(0.028)
Sinusitis	0.760***	0.854***	-0.902***	-0.904***
	(0.020)	(0.022)	(0.027)	(0.028)
Acute tonsilitis	1.392***	1.635***	-2.099***	-2.192***
	(0.024)	(0.028)	(0.029)	(0.031)
Other respiratory diagnoses	-1.291***	-1.402***	-0.234***	-0.281***
	(0.023)	(0.026)	(0.026)	(0.028)
Practice/list characteristics				
List length/100	0.015***	0.019***	0.031***	0.033***
	(0.004)	(0.004)	(0.005)	(0.005)
Open list	0.057***	0.065***	0.052***	0.047**
	(0.014)	(0.016)	(0.020)	(0.021)
Group practice	-0.077*	-0.123***	-0.140**	-0.135*

	(0.045)	(0.047)	(0.067)	(0.072)
Proportion females	0.167	0.220*	0.187	0.152
	(0.119)	(0.131)	(0.182)	(0.196)
Average age	0.008***	0.010***	-0.003	-0.003
	(0.002)	(0.002)	(0.003)	(0.003)
Proportion low income	0.423***	0.727***	-0.989***	-0.938***
	(0.153)	(0.174)	(0.260)	(0.275)
Month FE	Yes	Yes	Yes	Yes
Year FE	Yes	Yes	Yes	Yes
Municipality FE	Yes	Yes	Yes	Yes
Observations	9,977,798	6,972,642	1,487,309	1,162,589
AIC	6,696,189	4,874,135	1,773,398	1,398,187

Notes: *p<0.1; **p<0.05; ***p<0.01; FE – fixed effects. Robust standard errors clustered at the GP level are shown in parentheses. Estimation results for year, month and municipality dummies are omitted to save space and are available upon request.

According to Table S5, regular GPs paid by FFS and CAP were 11% more likely (odds ratio is equal to exp(0.104)≈1.11) to prescribe an antibiotic for RTI during the first/only contact of the infection episode than salaried regular GPs, and were 8% more likely (odds ratio is equal to exp(0.080)≈1.08) to prescribe an antibiotic for RTI for all contacts than salaried regular GPs. Further, regular GPs paid by FFS and CAP were 15% more likely (odds ratio is equal to exp(0.140)≈1.15) to choose a non-PcV antibiotic during the first/only contact than salaried regular GPs, and were 15% more likely (odds ratio is equal to exp(0.136)≈1.15) to choose a non-PcV antibiotic for all contacts than salaried regular GPs.

Table S5 Estimation results of the Models 1 and 2 with probit regression for regular GPs, log odds ratios

	Dependent variable:			
	Antibiotic prescribed (Model 1)		Non-PcV chosen (Model 2)	
	all RTI contacts	first (the only) contacts for RTI episodes	all RTI contacts	first (the only) contacts for RTI episodes
FFS+CAP	0.080***	0.104***	0.136***	0.140***
	(0.028)	(0.031)	(0.039)	(0.044)
GP characteristics				
Specialist	-0.019**	-0.031***	-0.103***	-0.114***
	(0.010)	(0.011)	(0.017)	(0.018)
Male	0.001	0.003	0.074***	0.072***
	(0.012)	(0.014)	(0.021)	(0.023)
Age	0.003***	0.004***	0.007***	0.008***
	(0.0004)	(0.0005)	(0.001)	(0.001)
Patient characteristics				
Age group 3-6	0.127***	0.124***	-0.073***	-0.054***
	(0.004)	(0.004)	(0.009)	(0.009)

Age group 7-12	0.133***	0.109***	-0.262***	-0.300***
	(0.005)	(0.005)	(0.013)	(0.014)
Age group 13-18	0.116***	0.119***	-0.296***	-0.355***
	(0.005)	(0.006)	(0.015)	(0.017)
Age group 19-25	0.190***	0.231***	-0.242***	-0.268***
	(0.006)	(0.006)	(0.015)	(0.016)
Age group 26-45	0.169***	0.229***	-0.112***	-0.136***
	(0.005)	(0.006)	(0.014)	(0.016)
Age group 46-65	0.173***	0.259***	0.130***	0.123***
	(0.006)	(0.006)	(0.015)	(0.016)
Age group 66-85	0.169***	0.274***	0.198***	0.194***
	(0.006)	(0.006)	(0.015)	(0.016)
Age group 86+	0.059***	0.157***	-0.003	0.044**
	(0.008)	(0.009)	(0.018)	(0.020)
Male	-0.013***	-0.014***	-0.042***	-0.044***
	(0.002)	(0.002)	(0.003)	(0.003)
Household income/100,000	0.002***	0.001**	0.003***	0.003***
	(0.001)	(0.001)	(0.001)	(0.001)
Patient is listed with the GP	0.007	0.011	0.079***	0.062**
	(0.014)	(0.016)	(0.026)	(0.027)
Contact characteristics				
Telephone contact	0.054***	0.077***	0.117***	0.045***
	(0.009)	(0.012)	(0.012)	(0.017)
Consultation	0.895***	1.062***	-0.373***	-0.319***
	(0.008)	(0.010)	(0.010)	(0.013)
Home visit	0.617***	0.753***	-0.266***	-0.268***
	(0.018)	(0.022)	(0.030)	(0.036)
Diagnosis				
Upper RT symptoms and infections	-0.709***	-0.766***	-0.505***	-0.536***
	(0.011)	(0.013)	(0.014)	(0.016)
Chronic bronchitis/ COPD	-0.799***	-0.888***	0.655***	0.718***
	(0.012)	(0.015)	(0.018)	(0.019)
Ear infection	0.295***	0.354***	-0.751***	-0.784***
	(0.013)	(0.015)	(0.016)	(0.018)
Pneumonia	0.304***	0.530***	-0.211***	-0.279***
	(0.012)	(0.015)	(0.015)	(0.017)
Sinusitis	0.443***	0.505***	-0.546***	-0.550***
	(0.011)	(0.013)	(0.016)	(0.017)
Acute tonsilitis	0.830***	0.975***	-1.269***	-1.318***
	(0.014)	(0.016)	(0.017)	(0.018)
Other respiratory diagnoses	-0.703***	-0.779***	-0.138***	-0.168***
	(0.012)	(0.014)	(0.015)	(0.017)
Practice/list characteristics				
List length/100	0.008***	0.010***	0.019***	0.020***
	(0.002)	(0.002)	(0.003)	(0.003)
Open list	0.031***	0.034***	0.031***	0.029**
	(0.007)	(0.008)	(0.012)	(0.013)
Group practice	-0.046*	-0.071***	-0.082**	-0.080*
	(0.024)	(0.026)	(0.040)	(0.044)

Proportion females	0.084	0.116	0.114	0.092
	(0.063)	(0.071)	(0.110)	(0.118)
Average age	0.004***	0.005***	-0.002	-0.002
	(0.001)	(0.001)	(0.002)	(0.002)
Proportion low income	0.221***	0.393***	-0.598***	-0.565***
	(0.082)	(0.093)	(0.158)	(0.167)
Month FE	Yes	Yes	Yes	Yes
Year FE	Yes	Yes	Yes	Yes
Municipality FE	Yes	Yes	Yes	Yes
Observations	9,977,798	6,972,642	1,487,309	1,162,589
AIC	6,715,428	4,887,222	1,774,517	1,398,764

Notes: *p<0.1; **p<0.05; ***p<0.01; FE – fixed effects. Robust standard errors clustered at the GP level are shown in parentheses. Estimation results for year, month and municipality dummies are omitted to save space and are available upon request.

According to Table S6, locum GPs paid by FFS and CAP were 14% more likely (odds ratio is equal to exp(0.131)≈1.14) to choose a non-PcV antibiotic during the first/only contact than salaried locum GPs, and were 17% more likely (odds ratio is equal to exp(0.160)≈1.17) to choose a non-PcV antibiotic for all contacts than salaried locum GPs.

Table S6 Estimation results of the Models 1 and 2 with logit regression for locums, log odds ratios

	Dependent variable:			
	Antibiotic prescribed (Model 1)		Non-PcV chosen (Model 2)	
	all RTI contacts	first (the only) contacts for RTI episodes	all RTI contacts	first (the only) contacts for RTI episodes
FFS	0.018	0.052	0.160***	0.131**
	(0.032)	(0.035)	(0.053)	(0.057)
GP characteristics				
Specialist	-0.093**	-0.102**	-0.123**	-0.119*
	(0.042)	(0.047)	(0.060)	(0.063)
Male	-0.019	-0.024	0.067	0.065
	(0.024)	(0.027)	(0.043)	(0.046)
Age	0.013***	0.015***	0.022***	0.024***
	(0.001)	(0.001)	(0.002)	(0.002)
Patient characteristics				
Age group 3-6	0.216***	0.214***	-0.072**	-0.054
	(0.015)	(0.016)	(0.032)	(0.033)
Age group 7-12	0.152***	0.098***	-0.258***	-0.319***
	(0.018)	(0.020)	(0.040)	(0.042)
Age group 13-18	0.115***	0.110***	-0.269***	-0.371***
	(0.018)	(0.019)	(0.042)	(0.045)
Age group 19-25	0.206***	0.253***	-0.161***	-0.209***

	(0.018)	(0.019)	(0.041)	(0.044)
Age group 26-45	0.170***	0.240***	0.037	-0.003
	(0.016)	(0.018)	(0.039)	(0.041)
Age group 46-65	0.200***	0.310***	0.446***	0.434***
	(0.017)	(0.018)	(0.039)	(0.041)
Age group 66-85	0.204***	0.362***	0.606***	0.598***
	(0.018)	(0.019)	(0.041)	(0.043)
Age group 86+	-0.092***	0.056*	0.281***	0.361***
	(0.027)	(0.031)	(0.056)	(0.060)
Male	-0.045***	-0.039***	-0.092***	-0.092***
	(0.006)	(0.006)	(0.010)	(0.011)
Household income/100,000	0.005***	0.004***	0.007***	0.006***
	(0.001)	(0.001)	(0.002)	(0.002)
Contact characteristics				
Telephone contact	0.396***	0.342***	0.395***	0.333***
	(0.035)	(0.046)	(0.051)	(0.081)
Consultation	1.799***	2.206***	-0.830***	-0.713***
	(0.028)	(0.037)	(0.043)	(0.067)
Home visit	1.218***	1.400***	-0.579***	-0.544***
	(0.074)	(0.091)	(0.115)	(0.141)
Diagnosis				
Upper RT symptoms and infections	-1.122***	-1.165***	-0.745***	-0.793***
	(0.029)	(0.032)	(0.037)	(0.041)
Chronic bronchitis/ COPD	-1.074***	-1.044***	1.690***	1.779***
	(0.034)	(0.040)	(0.072)	(0.075)
Ear infection	0.927***	1.071***	-1.218***	-1.274***
	(0.033)	(0.037)	(0.042)	(0.046)
Pneumonia	1.034***	1.399***	-0.301***	-0.427***
	(0.033)	(0.038)	(0.039)	(0.043)
Sinusitis	0.776***	0.829***	-0.945***	-0.942***
	(0.030)	(0.034)	(0.051)	(0.057)
Acute tonsilitis	1.808***	2.088***	-1.927***	-1.983***
	(0.034)	(0.039)	(0.043)	(0.047)
Other respiratory diagnoses	-1.105***	-1.218***	-0.124***	-0.207***
	(0.038)	(0.043)	(0.041)	(0.046)
Practice/list characteristics				
List length/100	0.009***	0.009**	0.008	0.010
	(0.003)	(0.004)	(0.006)	(0.006)
Open list	0.043***	0.036**	0.019	0.030
	(0.015)	(0.017)	(0.027)	(0.029)
Group practice	-0.021	-0.073	-0.160*	-0.185*
	(0.060)	(0.069)	(0.097)	(0.106)
Proportion females	-0.058	-0.067	-0.080	-0.074
	(0.077)	(0.084)	(0.151)	(0.151)
Average age	0.008***	0.009***	0.002	0.004
	(0.002)	(0.002)	(0.003)	(0.003)
Proportion low income	0.103	0.325*	-0.396	-0.258
	(0.166)	(0.183)	(0.297)	(0.303)
Month FE	Yes	Yes	Yes	Yes

Year FE	Yes	Yes	Yes	Yes
Municipality FE	Yes	Yes	Yes	Yes
Observations	1,520,168	1,164,473	234,614	193,672
AIC	1,037,853	808,307	278,550	226,851

Notes: *p<0.1; **p<0.05; ***p<0.01; FE – fixed effects. Robust standard errors clustered at the GP level are shown in parentheses. Estimation results for year, month and municipality dummies are omitted to save space and are available upon request.

According to Table S7, locum GPs paid by FFS and CAP were 8% more likely (odds ratio is equal to exp(0.078)≈1.08) to choose a non-PcV antibiotic during the first/only contact than salaried locum GPs, and were 10% more likely (odds ratio is equal to exp(0.096)≈1.10) to choose a non-PcV antibiotic for all contacts than salaried locum GPs.

Table S7 Estimation results of the Models L1 and L2 with probit regression for locums, log odds ratios

	Dependent variable:			
	Antibiotic prescribed (Model 1)		Non-PcV chosen (Model 2)	
	all RTI contacts	first (the only) contacts for RTI episodes	all RTI contacts	first (the only) contacts for RTI episodes
FFS	0.010	0.028	0.096***	0.078**
	(0.017)	(0.019)	(0.032)	(0.034)
GP characteristics				
Specialist	-0.053**	-0.056**	-0.075**	-0.073*
	(0.023)	(0.026)	(0.036)	(0.038)
Male	-0.008	-0.010	0.040	0.038
	(0.013)	(0.015)	(0.025)	(0.027)
Age	0.007***	0.008***	0.013***	0.014***
	(0.001)	(0.001)	(0.001)	(0.001)
Patient characteristics				
Age group 3-6	0.130***	0.131***	-0.041**	-0.030
	(0.008)	(0.009)	(0.019)	(0.020)
Age group 7-12	0.091***	0.062***	-0.160***	-0.195***
	(0.010)	(0.011)	(0.024)	(0.025)
Age group 13-18	0.069***	0.068***	-0.164***	-0.224***
	(0.010)	(0.010)	(0.025)	(0.027)
Age group 19-25	0.120***	0.147***	-0.101***	-0.130***
	(0.010)	(0.010)	(0.025)	(0.026)
Age group 26-45	0.097***	0.136***	0.018	-0.007
	(0.009)	(0.009)	(0.023)	(0.025)
Age group 46-65	0.109***	0.169***	0.269***	0.262***
	(0.009)	(0.010)	(0.023)	(0.025)
Age group 66-85	0.113***	0.199***	0.364***	0.362***
	(0.010)	(0.010)	(0.025)	(0.026)
Age group 86+	-0.041***	0.042**	0.165***	0.217***

	(0.014)	(0.017)	(0.034)	(0.037)
Male	-0.026***	-0.022***	-0.056***	-0.055***
	(0.003)	(0.004)	(0.006)	(0.007)
Household income/100,000	0.003***	0.002***	0.004***	0.004***
	(0.001)	(0.001)	(0.001)	(0.001)
Contact characteristics				
Telephone contact	0.194***	0.160***	0.229***	0.195***
	(0.017)	(0.023)	(0.031)	(0.049)
Consultation	0.899***	1.095***	-0.497***	-0.425***
	(0.014)	(0.018)	(0.026)	(0.041)
Home visit	0.606***	0.693***	-0.348***	-0.324***
	(0.039)	(0.049)	(0.069)	(0.085)
Diagnosis				
Upper RT symptoms and infections	-0.604***	-0.634***	-0.460***	-0.491***
	(0.016)	(0.019)	(0.022)	(0.025)
Chronic bronchitis/ COPD	-0.570***	-0.559***	0.909***	0.966***
	(0.019)	(0.022)	(0.039)	(0.040)
Ear infection	0.557***	0.643***	-0.750***	-0.782***
	(0.019)	(0.022)	(0.025)	(0.028)
Pneumonia	0.607***	0.834***	-0.184***	-0.264***
	(0.019)	(0.022)	(0.024)	(0.026)
Sinusitis	0.458***	0.497***	-0.580***	-0.579***
	(0.018)	(0.020)	(0.031)	(0.035)
Acute tonsilitis	1.087***	1.253***	-1.154***	-1.180***
	(0.020)	(0.023)	(0.025)	(0.027)
Other respiratory diagnoses	-0.586***	-0.655***	-0.074***	-0.127***
	(0.020)	(0.023)	(0.025)	(0.028)
Practice/list characteristics				
List length/100	0.005***	0.005**	0.005	0.006*
	(0.002)	(0.002)	(0.004)	(0.004)
Open list	0.022***	0.019**	0.011	0.018
	(0.008)	(0.009)	(0.016)	(0.017)
Group practice	-0.014	-0.043	-0.094	-0.110*
	(0.034)	(0.038)	(0.057)	(0.063)
Proportion females	-0.026	-0.031	-0.048	-0.043
	(0.043)	(0.046)	(0.090)	(0.089)
Average age	0.005***	0.005***	0.001	0.002
	(0.001)	(0.001)	(0.002)	(0.002)
Proportion low income	0.052	0.174*	-0.245	-0.158
	(0.092)	(0.100)	(0.178)	(0.181)
Month FE	Yes	Yes	Yes	Yes
Year FE	Yes	Yes	Yes	Yes
Municipality FE	Yes	Yes	Yes	Yes
Observations	1,520,168	1,164,473	234,614	193,672
AIC	1,039,894	809,794	278,745	226,975

Notes: *p<0.1; **p<0.05; ***p<0.01; FE – fixed effects. Robust standard errors clustered at the GP level are shown in parentheses. Estimation results for year, month and municipality dummies are omitted to save space and are available upon request.

GPs switching remuneration scheme

Table S8 presents the OLS estimation results for GPs switching the remuneration type on the datasets including the first or the only contact of each RTI episode. Estimation results of the probit and logit models are consistent with OLS and are available upon request.

Table S8 Estimation results for GPs switching the remuneration type with Linear Probability Model (OLS) on the datasets for the first or the only contact during each RTI episode

	Dependent variable:			
	Antibiotic is prescribed		Non-PcV antibiotic is chosen	
	Regular GPs	Locums	Regular GPs	Locums
FFS	0.020***	0.001	0.048**	-0.009
	(0.007)	(0.004)	(0.022)	(0.010)
GP characteristics				
Specialist	-0.010	0.001	-0.006	-0.030
	(0.006)	(0.011)	(0.019)	(0.023)
Patient characteristics				
Age group 3-6	0.014**	0.028***	-0.006	-0.028**
	-0.007	-0.004	-0.024	-0.013
Age group 7-12	0.027***	0.012**	-0.04	-0.052***
	-0.009	-0.005	-0.025	-0.017
Age group 13-18	0.032***	0.023***	-0.034	-0.069***
	-0.01	-0.005	-0.027	-0.019
Age group 19-25	0.048***	0.032***	-0.026	-0.046**
	-0.009	-0.005	-0.027	-0.019
Age group 26-45	0.052***	0.034***	0.039	-0.008
	-0.009	-0.005	-0.026	-0.018
Age group 46-65	0.053***	0.041***	0.138***	0.092***
	-0.009	-0.005	-0.024	-0.018
Age group 66-85	0.046***	0.045***	0.127***	0.128***
	-0.008	-0.005	-0.025	-0.018
Age group 86+	0.018*	0.006	0.116***	0.098***
	-0.009	-0.006	-0.035	-0.027
Male	-0.001	-0.005***	-0.013	-0.027***
	-0.002	-0.002	-0.009	-0.005
Household income/100,000	0.001*	0.0002*	-0.001	0.002*
	-0.0005	-0.0001	-0.002	-0.001
Contact characteristics				
Telephone contact	0.016*	0.013***	-0.061*	0.096***
	-0.009	-0.005	-0.032	-0.033
Consultation	0.140***	0.154***	-0.103***	-0.122***
	-0.011	-0.005	-0.022	-0.022
Home visit	0.058***	0.033**	-0.02	-0.067

	-0.019	-0.015	-0.054	-0.067
Diagnosis				
Upper RT symptoms and infections	-0.182***	-0.184***	-0.150***	-0.142***
	-0.023	-0.018	-0.018	-0.023
Chronic bronchitis/ COPD	-0.187***	-0.153***	0.155***	0.275***
	-0.024	-0.019	-0.029	-0.026
Ear infection	0.096***	0.183***	-0.269***	-0.254***
	-0.026	-0.021	-0.031	-0.023
Pneumonia	0.122***	0.242***	-0.056**	-0.024
	-0.025	-0.02	-0.025	-0.027
Sinusitis	0.124***	0.144***	-0.181***	-0.141***
	-0.023	-0.017	-0.031	-0.042
Acute tonsilitis	0.307***	0.389***	-0.376***	-0.315***
	-0.033	-0.021	-0.027	-0.021
Other respiratory diagnoses	-0.190***	-0.183***	-0.028	0.002
	-0.024	-0.019	-0.025	-0.025
Practice/list characteristics				
List length/100	-0.0002	0.001**	0.002	-0.0002
	-0.002	-0.0004	-0.008	-0.001
Open list	-0.003	0.006**	-0.015	0.007
	-0.005	-0.003	-0.017	-0.008
Group practice	0.018*	-0.001	-0.007	0.026*
	-0.01	-0.008	-0.033	-0.015
Proportion females	0.055	-0.01	0.214	-0.011
	-0.069	-0.014	-0.335	-0.038
Average age	-0.001	0.0003	-0.007	0.001
	-0.002	-0.0003	-0.007	-0.001
Proportion low income	-0.058	-0.087***	0.056	-0.084
	-0.089	-0.023	-0.306	-0.053
Month FE	Yes	Yes	Yes	Yes
Year FE	Yes	Yes	Yes	Yes
Municipality FE	Yes	Yes	Yes	Yes
GP FE	Yes	Yes	Yes	Yes
Observations	101,980	196,624	14,585	34,856
R2	0.217	0.263	0.277	0.266

Notes: *p<0.1; **p<0.05; ***p<0.01; FE – fixed effects. Robust standard errors clustered at the GP level are shown in parentheses. Estimation results for year, month and municipality dummies are omitted to save space and are available upon request.
